# Prevalence of depression among Chinese university students: a systematic review and meta-analysis

**DOI:** 10.1038/s41598-020-72998-1

**Published:** 2020-09-28

**Authors:** Li Gao, Yuanchen Xie, Chunhua Jia, Wei Wang

**Affiliations:** 1 Beijing University of Chinese Medicine, Beijing, 100029 China; 2 St Michael’s Hospital, University of Toronto, Toronto, M5B 1W8 Canada; 3 Zhengzhou University, Zhengzhou, 450052 China

**Keywords:** Quality of life, Psychology

## Abstract

Estimates of the depression prevalence among Chinese university students vary considerably across studies. This systematic review and meta-analysis aimed to comprehensively analyze the depression prevalence among Chinese university students. We searched four electronic databases with the search terms of depression, China, university student, and questionnaire. Studies reporting depression among Chinese university students were included in the analysis. Two reviewers independently extracted the data and assessed the qualities of the studies. The package of “meta” in R Foundation for Statistical Computing was used to calculate an overall proportion in a random-effects model with 95% confidence intervals. Subgroup analysis was conducted to analyze the influencing factors on the depression prevalence. Any conflict in the data analysis was discussed by all the reviewers. A total of 113 studies were included in the meta-analysis. The overall prevalence of depression among Chinese university students was shown to be 28.4% (n = 185,787), with 95%CI from 25.7 to 31.2%. The overall depression prevalence among Chinese university students was still relatively high. More efforts need to be done to provide better mental healthcare to university students in China.

## Introduction

Depression is an emotional disorder that is common among university students^1^. It causes a persistent sad feeling for a relatively long period and may even lead to suicidal ideation^2–4^. Depression in university students may not only affect their academic performances but also may hinder their future success. Studies have shown that the depression prevalence among university students was relatively high^5,6^. According to the hypotheses of different studies, university students are vulnerable to depression, as they are facing more challenges, such as continuing study pressure, changes in residence place and lifestyle, economic pressure, or employment pressure, etc.^7–9^.

In recent years, many scholars have conducted surveys to analyze the psychological changes among Chinese university students, such as depression, anxiety, suicidal ideation. Dong et al.^10^ conducted a cross-sectional survey to analyze the depressive symptoms among college students in Liaoning, China, the result showed the depression prevalence among 1362 students was 32.8%. Hou et al.^11^ conducted a cross-sectional survey with 4119 Chinese university students and found the depression prevalence was 9.7%. Tang et al.^12^ conducted a cross-sectional survey among 5972 Chinese undergraduates and found 1,170 students were classified as mild to high depression, and the prevalence was 19.6%. It can be seen from the above discussion that the estimates of the depression prevalence varied significantly across studies.

Meta-analysis is a reliable tool to estimate the depression prevalence among university students. Ibrahim et al.^1^ analyzed 24 studies published between 1990 and 2010 and pooled the depression prevalence ranged from 10 to 85% with an overall prevalence of 30.6%. Rotenstein et al.^13^ conducted a meta-analysis to estimate the prevalence of depression among medical students and found that the prevalence rate ranged widely (from 1.4 to 73.5%).

In terms of depression prevalence among Chinese university students, Lei et al.^14^ conducted a meta-analysis of 39 studies, which were published between 1997 and 2015. The result showed that the overall estimate was 23.8%, while the depression prevalence ranged from 3.0 to 80.6% in individual studies. Recently, a number of new studies have been published to estimate depression prevalence among Chinese university students. In addition, the work of Lei et al.^14^ did not consider the depression screening method and cutoff score as an influencing factor in the analysis. The different screening methods will result in an estimate of different depression prevalence, and a higher cutoff score is usually accompanied by a lower depression prevalence. Therefore, a stratified meta-analyses of screening method and cutoff score is an important way to characterize the differences.

As reliably estimating the prevalence of depression is an important step to making efforts to provide better mental healthcare to university students, it is necessary to update the meta-analysis to better understand the depression prevalence in recent years. In this study, a systematic review and meta-analysis was conducted to comprehensively analyze the depression prevalence among Chinese university students.

## Results

### Description of the included studies

**Figure 1:**
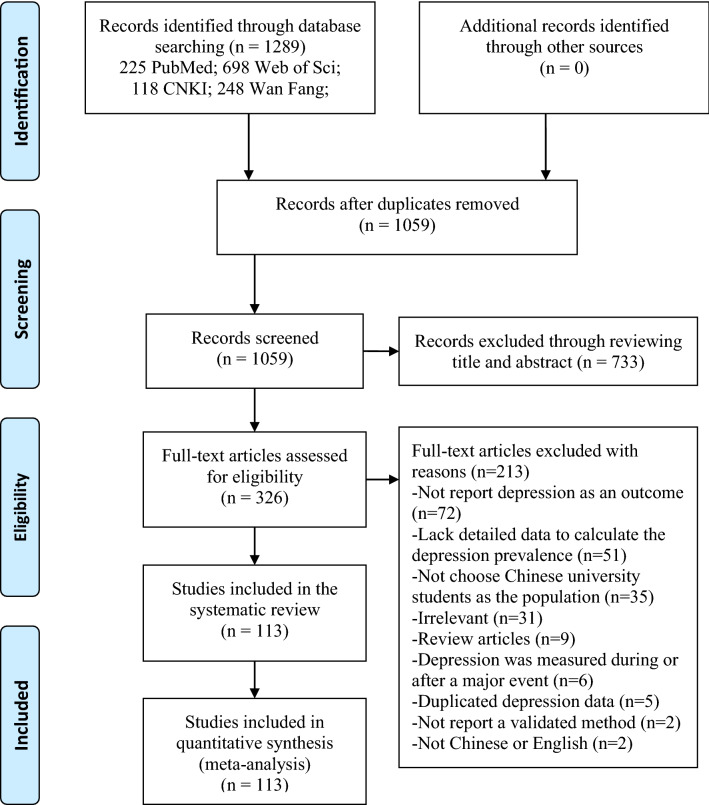
Flowchart of the selection process.

In this meta-analysis, we identified 1289 potentially eligible studies (923 in English and 366 in Chinese). After removing repeated publications, 1059 studies were left to be screened. Titles and abstracts of all studies were screened, 733 studies were excluded for irrelevance. The remaining 326 studies were screened by reading the full text, and 213 studies were excluded for reasons below: 72 studies did not report depression as an outcome; 51 studies did not report detailed data of the depression to calculate the depression prevalence; 35 studies did not choose Chinese university students as the population, or include non-Chinese university students in the study population, and cannot distinguish depression data of the Chinese university students; 31 studies were irrelevant; 9 studies were review articles; 6 studies were measuring depression during or after a major event; 5 studies used depression data of the population which has been published before; 2 studies did not report a validated method to screen depression; 2 studies were not written in English or Chinese. At last, 113 studies were included in the meta-analysis and detailed information on the selection process is shown in Fig. 1. References of the 113 studies are shown in the online Appendix.

Table 1 shows the main characteristics of the 113 included studies. 48 studies were written in English and 65 studies were written in Chinese. The whole population was 185,787 Chinese university students, with an average population of approximately 1,644 students per study. In total, 46,421 students were screened as depression. The depression prevalence of the included studies ranged from 3.0 to 80.6%. For the studies reported the number of men and women, the male proportion was 46.6% (Male 84,488/Total 181,253). There were 26 categories of method and cutoff score to screen depression. The most commonly used screening method and cutoff score was SDS ≥ 50, 26 studies screened depression with this method. There were 10 categories of method and cutoff score that were used only once. Depression prevalence and 95% CI for each study was calculated and shown in the Table 1. The most-reported influencing factor on depression was gender, followed by family origin. Qualities of the studies are shown in online Appendix-Table 1. A total of 8 studies^15–22^ were assessed at a high risk of bias (modified Newcastle–Ottawa scale < 3 points).

**Table 1:** Main characteristics of the 113 included studies.

Study ID	Number of Depressed/Total students	Prevalence, % (95% CI)	Language	Age, years	Male proportion	Screening method and cutoff score	Influencing factors	Location
Cai (2017)	421/1327	31.7 (29.2–34.3)	EN	19.8 ± 1.3	100.0%	SDS ≥ 53	S	Hubei
Chai (2011)	400/1681	23.8 (21.8–25.9)	CH	18–22	53.5%	SDS ≥ 53	G, F	Hubei
Chan (1992)	15/95	15.8 (9.1–24.7)	EN	19.6 ± 1.4	67.4%	BDI > 18	NR	Hongkong
Chang (2011)	96/255	37.6 (31.7–43.9)	EN	NR	39.2%	TDQ	NR	Taiwan
Chen (2013)	617/5245	11.8 (10.9–12.7)	EN	21.3 ± 2.2	48.9%	BDI ≥ 14	G, A, EG, M	Heilongjiang
Chen (2015)	204/625	32.6 (29.0–36.5)	EN	17.4 ± 1.0	5.4%	ADI ≥ 8	NR	Taiwan
Chen (2016)	141/501	28.1 (24.2–32.3)	CH	18–25	58.1%	SDS ≥ 50	NR	Beijing
Cheung (2016)	232/661	35.1 (31.5–38.9)	EN	18–30	27.5%	DASS-21 ≥ 10	G, A, R, S	Hongkong
Chou (2018)	89/324	27.5 (22.7–32.7)	EN	22.1 ± 1.8	47.8%	BDI-II ≥ 14	NR	NR
Deng (2011)	650/2768	23.5 (21.9–25.1)	EN	19.4 ± 1.1	0.0%	SDS ≥ 53	NR	Anhui
Dong (2019)	447/1362	32.8 (30.3–35.4)	EN	18–26	53.9%	CES-D > 16	NR	Liaoning
Du (1999)	564/1277	44.2 (41.4–46.9)	CH	20.7 ± 1.4	60.6%	BDI ≥ 10	NR	Shandong
Fang (2017)	885/1475	60.0 (57.4–62.5)	CH	NR	NR	SDS ≥ 50	NR	Multiple
Feng (2005)	79/480	16.5 (13.3–20.1)	CH	20.2 ± 1.5	52.9%	SDS ≥ 40	NR	Shandong
Feng (2014)	117/1106	10.6 (8.8–12.5)	EN	18.9 ± 0.9	57.4%	SDS ≥ 53	NR	Hubei
Fu (2010)	321/631	50.9 (46.9–54.8)	CH	NR	51.7%	SDS ≥ 50	NR	Jilin
Gao (2008)	79/253	31.2 (25.6–37.3)	CH	20.3 ± 1.1	30.0%	CES-D ≥ 20	NR	Ningxia
Gao (2018)	126/730	17.3 (14.6–20.2)	EN	20.5 ± 1.4	30.0%	BDI-II ≥ 14	NR	Jilin
Guo (2013)	329/745	44.2 (40.6–47.8)	CH	19.4 ± 1.1	3.9%	SDS ≥ 50	F	Hunan
Guo (2019)	139/306	45.4 (39.8–51.2)	CH	19–26	49.3%	SDS ≥ 50	G	Fujian
Guo (2020)	697/3278	21.3 (19.9–22.7)	EN	18.4 ± 1.3	28.3%	SDS ≥ 53	NR	Liaoning
Hall (2018)	55/101	54.5 (44.2–64.4)	EN	22.3 ± 2.6	30.7%	DASS-21 ≥ 14	NR	Multiple
Han (2011)	80/381	21.0 (17.0–25.4)	CH	NR	NR	SDS ≥ 53	NR	Shanghai
Han (2015)	70/843	8.3 (6.5–10.4)	CH	NR	29.5%	SDS	NR	Liaoning
Han (2018)	265/788	33.6 (30.3–37.0)	EN	19.6 ± 1.0	51.9%	SDS ≥ 53	L	Sichuan
He (2015)	398/1186	33.6 (30.9–36.3)	CH	15–25	36.4%	SDS ≥ 50	G, A, O, EG, D, R	Beijing
Hou (2014)	23/978	2.4 (1.5–3.5)	CH	NR	7.4%	CCSMHS	NR	Liaoning
Hou H (2018)	401/4119	9.7 (8.8–10.7)	EN	Ave 19	27.0%	SCL-90 ≥ 2	G, F, A, M	Multiple
Hou Y (2018)	333/2519	13.2 (11.9–14.6)	CH	21.9 ± 1.3	38.5%	SDS ≥ 53	G, O, L, EF, EM	Guangdong
Hu (2012)	101/307	32.9 (27.7–38.5)	CH	18–24	47.6%	SDS ≥ 53	G, F, A	Multiple
Hua (2008)	23/98	23.5 (15.5–33.1)	CH	NR	62.2%	SDS ≥ 50	G	Shanghai
Jiang (2011)	1893/6009	31.5 (30.3–32.7)	CH	NR	53.2%	SDS ≥ 50	G, F, A, L, EF, EM	Anhui
Jin (2014)	395/1095	36.1 (33.2–39.0)	CH	NR	52.1%	SDS ≥ 50	G, F	Jiangsu
Kang (2017)	102/519	19.7 (16.3–23.3)	CH	22.5 ± 1.5	45.1%	SDS ≥ 50	NR	Jilin
Lei (2018)	495/7842	6.3 (5.8–6.9)	CH	24.2 ± 1.0	63.7%	CES-D ≥ 28	NR	Multiple
Li (2018)	203/629	32.3 (28.6–36.1)	CH	19.7 ± 1.6	42.9%	CES-D ≥ 16	G, F, O	Guizhou
Lin (2018)	120/913	13.1 (11.0–15.5)	CH	NR	44.0%	BDI ≥ 14	G, A	Hubei
Liu (1997)	97/560	17.3 (14.3–20.7)	CH	20.9 ± 1.8	57.5%	SDS ≥ 50	NR	NR
Liu (2011)	54/185	29.2 (22.8–36.3)	CH	18.7 ± 2.2	51.4%	SDS ≥ 50	NR	Shandong
Liu (2014)	312/804	38.8 (35.4–42.3)	CH	17–24	45.1%	CES-D ≥ 16	G, A, D	Hunan
Liu (2015)	137/755	18.1 (15.5–21.1)	CH	18–25	17.5%	CES-D ≥ 20	G, F, O, EG, R	NR
Liu (2017)	198/1006	19.7 (17.3–22.3)	CH	17–26	45.3%	SDS ≥ 50	NR	Hunan
Liu (2019)	490/1401	35.0 (32.5–37.5)	EN	NR	53.6%	DASS-21 ≥ 10	NR	Beijing
Liu (2020)	300/1505	19.9 (17.9–22.0)	EN	NR	39.5%	SCL-90 > 2	L	Shandong
Lu (2015)	687/1048	65.6 (62.6–68.4)	EN	18.6 ± 0.9	66.3%	PHQ-9 ≥ 5	G, F, EG, R	Shanghai
Luo (2004)	152/275	55.3 (49.2–61.2)	CH	20.8 ± 1.1	18.2%	CES-D ≥ 16	NR	Zhejiang
Ma (2019)	152/960	15.8 (13.6–18.3)	CH	18.3 ± 0.9	57.0%	SDS ≥ 53	NR	Hebei
Niu (2010)	132/609	21.7 (18.5–25.2)	CH	NR	52.9%	SDS ≥ 53	G	Shandong
Pan (2016)	1751/8819	19.9 (19.0–20.7)	EN	20.7 ± 1.6	38.7%	BDI ≥ 14	G, F, EG, M, EF	Multiple
Peng (2010)	653/1178	55.4 (52.5–58.3)	EN	NR	66.7%	CES-D ≥ 16	G, F	Hunan
Shao (2020)	1183/2057	57.5 (55.3–59.7)	EN	19.8 ± 1.2	29.3%	SDS ≥ 50	G, F, A, O, EG	Chongqing
Shen (2016)	594/1931	30.8 (28.7–32.9)	CH	20.3 ± 1.1	32.5%	SDS ≥ 53	G, O, M, D	Jiangsu
Shi (2016)	1954/2925	66.8 (65.1–68.5)	EN	21.7 ± 2.0	35.1%	CES-D ≥ 16	NR	Liaoning
Sobowale (2014)	226/348	64.9 (59.7–70.0)	EN	NR	NR	PHQ-9 ≥ 5	NR	Hubei
Song (2008)	551/1677	32.9 (30.6–35.2)	EN	18.2 ± 1.5	51.3%	CES-D ≥ 16	G	Multiple
Sun (2008)	53/1171	4.5 (3.4–5.9)	CH	17–24	45.5%	SCL-90 > 2	NR	Henan
Sun (2011)	1699/10,140	16.8 (16.0–17.5)	EN	19.6 ± 1.3	46.2%	BDI ≥ 10	G, A	Anhui
Sun (2013)	510/690	73.9 (70.5–77.2)	CH	21.0 ± 2.0	80.9%	CES-D ≥ 16	G, F	NR
Sun (2017)	708/5989	11.8 (11.0–12.7)	EN	20.9 ± 0.6	53.4%	BDI-II ≥ 14	G, F, A	Hubei
Tan (2012)	89/588	15.1 (12.3–18.3)	CH	17–23	50.0%	SDS ≥ 53	G	Jiangxi
Tang F (2018)	1170/5972	19.6 (18.6–20.6)	EN	20.9 ± 0.6	53.4%	SCL-90-R ≥ 1	NR	Hubei
Tang W (2018)	908/2563	35.4 (33.6–37.3)	EN	18.3 ± 0.9	65.5%	PHQ-9 ≥ 5	G, F, O	Sichuan
Tong (2016)	363/1872	19.4 (17.6–21.3)	EN	19.1 ± 1.3	52.4%	SCL-90-R ≥ 2	NR	Shanghai
Wang (2007)	396/1440	27.5 (25.2–29.9)	CH	20.8 ± 2.3	NR	SDS ≥ 50	NR	NR
Wang (2011)	45/930	4.8 (3.6–6.4)	CH	17.1 ± 2.3	56.3%	SCL-90 ≥ 2	NR	Liaoning
Wang (2012)	96/457	21.0 (17.4–25)	CH	20.3 ± 1.9	26.5%	BDI ≥ 14	NR	Hebei
Wang (2013)	438/1687	26.0 (23.9–28.1)	CH	19.8 ± 1.1	45.3%	SDS ≥ 53	G	Anhui
Wang L (2019)	259/2198	11.8 (10.5–13.2)	EN	Ave 20.5	43.5%	DASS-21	NR	Anhui
Wang M (2019)	1595/6284	25.4 (24.3–26.5)	EN	15–25	52.7%	CES-D ≥ 16	NR	Jilin
Wang Z (2019)	236/667	35.4 (31.8–39.1)	CH	19.9 ± 2.9	0.0%	SDS ≥ 53	NR	Multiple
Wei (2011)	151/391	38.6 (33.8–43.6)	CH	20.0 ± 2.0	42.2%	SDS ≥ 50	NR	Fujian
Wu (2007)	303/1334	22.7 (20.5–25.1)	CH	NR	0.0%	CES-D ≥ 20	A	Multiple
Wu (2015)	754/4747	15.9 (14.9–17.0)	EN	19.2 ± 1.4	41.6%	CES-D ≥ 16	G	Anhui
Wu (2016)	392/2521	15.5 (14.2–17.0)	EN	18.4 ± 1.0	47.1%	CES-D ≥ 16	NR	Anhui
Xi (2010)	84/402	20.9 (17.0–25.2)	CH	NR	NR	SDS ≥ 53	NR	Hebei
Xiao (2006)	218/558	39.1 (35.0–43.3)	CH	16–25	44.3%	SDS > 50	G, A	NR
Xiao (2016)	105/520	20.2 (16.8–23.9)	EN	20.1 ± 1.1	44.2%	CES-D ≥ 16	NR	Hunan
Xu (2002)	31/211	14.7 (10.2–20.2)	CH	Ave 19.7	38.9%	SDS ≥ 50	G	NR
Xu (2003)	381/1750	21.8 (19.9–23.8)	CH	Ave 21.8	48.3%	SDS ≥ 40	NR	Hebei
Xu (2014)	175/763	22.9 (20.0–26.1)	EN	17–22	13.4%	CES-D ≥ 16	G, F, A, O, EF, EM	Guangdong
Xu (2016)	566/1907	29.7 (27.6–31.8)	EN	Ave 19.5	46.7%	CES-D ≥ 16	NR	Guangdong
Xu (2020)	2080/4624	45.0 (43.5–46.4)	CH	19.9 ± 1.3	44.5%	PHQ-9 ≥ 5	NR	Multiple
Yang (2008)	98/222	44.1 (37.5–50.9)	CH	Ave 19.5	NR	SDS ≥ 50	NR	NR
Yang C (2013)	687/1372	50.1 (47.4–52.8)	CH	20.7 ± 1.4	33.5%	SDS ≥ 53	NR	Henan
Yang H (2013)	815/1972	41.3 (39.1–43.5)	CH	NR	31.2%	CES-D ≥ 16	G, F, EG	NR
Yang M (2007)	47/108	43.5 (34.0–53.4)	CH	NR	37.0%	CES-D ≥ 16	NR	NR
Yang X (2007)	113/3744	3.0 (2.5–3.6)	CH	16–23	75.5%	SDS ≥ 50	G, F	NR
Ye (2016)	820/2422	33.9 (32.0–35.8)	EN	19.7 ± 1.2	59.2%	SDS ≥ 53	NR	Hubei
Yen (2011)	235/2262	10.4 (9.2–11.7)	EN	21.0 ± 1.8	47.5%	CES-D ≥ 28	NR	Taiwan
Yu (2011)	140/600	23.3 (20.0–26.9)	CH	20.3 ± 1.2	12.5%	SDS ≥ 50	G	Shandong
Yu (2015)	538/4582	11.7 (10.8–12.7)	EN	20.8 ± 1.5	50.2%	BDI ≥ 14	NR	Heilongjiang
Zeng (2003)	40/302	13.2 (9.6–17.6)	CH	21.1 ± 1.1	70.9%	SDS ≥ 50	NR	NR
Zeng (2006)	205/408	50.2 (45.3–55.2)	CH	NR	55.4%	SDS ≥ 40	G, M	NR
Zeng (2019)	156/544	28.7 (24.9–32.7)	EN	20.2 ± 1.2	2.6%	DASS-21	NR	Sichuan
Zhai (2005)	114/509	22.4 (18.8–26.3)	CH	20.8 ± 1.3	41.1%	SDS ≥ 50	G	NR
Zhang (2004)	49/877	5.6 (4.2–7.3)	CH	20.5 ± 1.3	39.1%	SCL-90 > 2	NR	Hunan
Zhang (2005)	613/1351	45.4 (42.7–48.1)	CH	20.0 ± 1.0	42.7%	SDS ≥ 50	G, A	Henan
Zhang (2006)	693/860	80.6 (77.8–83.2)	CH	21.5 ± 2.3	47.4%	DSI ≥ 0.5	NR	Guangdong
Zhang (2015)	467/1853	25.2 (23.2–27.2)	CH	21.3 ± 1.8	30.8%	CES-D ≥ 16	NR	Multiple
Zhang (2018)	112/468	23.9 (20.1–28.1)	EN	19.3 ± 1.1	41.7%	DASS-21 ≥ 10	NR	Macao
Zhang (2020)	25/265	9.4 (6.2–13.6)	EN	18.9 ± 0.7	49.1%	SDS ≥ 53	NR	Zhejiang
Zhao (2018)	136/298	45.6 (39.9–51.5)	EN	20.3 ± 3.3	28.2%	CES-D ≥ 16	NR	Multiple
Zheng (2008)	784/1274	61.5 (58.8–64.2)	CH	19.1 ± 1.3	25.9%	SDS > 50	NR	NR
Zheng (2016)	101/324	31.2 (26.2–36.5)	CH	20.0 ± 1.9	46.3%	SDS ≥ 50	G, F, A, M, D, S	Hubei
Zhong (2011)	290/742	39.1 (35.6–42.7)	CH	20.7 ± 1.6	68.6%	HAMD ≥ 8	G, F, A, D	NR
Zhou (2003)	71/176	40.3 (33.0–48.0)	CH	21.4 ± 0.9	50.0%	SDS ≥ 40	G	Guangdong
Zhou (2009)	70/1179	5.9 (4.7–7.4)	CH	17–26	36.6%	BDI > 18	G, F, A	Hubei
Zhou (2018)	90/1159	7.8 (6.3–9.5)	EN	NR	36.2%	SCL-90 ≥ 2	NR	Jilin
Zhu (2019)	3648/10,174	35.9 (34.9–36.8)	EN	19.8 ± 0.9	61.8%	SDS ≥ 50	NR	Liaoning
Zong (2010)	56/266	21.1 (16.3–26.5)	EN	NR	NR	BDI ≥ 14	NR	Beijing
Zou (2007)	73/434	16.8 (13.4–20.7)	CH	20.0 ± 1.1	45.4%	SDS ≥ 50	NR	Shandong
Zou & Sun (2018)	39/582	6.7 (4.8–9.0)	EN	22.4 ± 1.2	100.0%	DASS-21 ≥ 10	NR	Chongqing
Zou & Wang (2018)	63/587	10.7 (8.3–13.5)	EN	20.3 ± 1.1	100.0%	SDS ≥ 53	NR	Chongqing

Ave, Average; CH, Chinese; EN, English; NR, not reported; ADI, Adolescent Depression Inventory; BDI, Beck Depression Inventory; CCSMHS, Chinese College Student Mental Health Scale; CES-D, Center for Epidemiologic Studies Depression Scale; DASS-21, Depression Anxiety Stress Scale 21; HAMD, Hamilton Rating Scale for Depression; PHQ-9, Patient Health Questionnaire-9; SCL-90, Symptom Checklist 90; SCL-90-R, Symptom Checklist 90 Revised; SDS, Self-Rating Depression Scale; TDQ, Taiwanese Depression Questionnaire; G, Gender; F, Family origin; A, Academic grade; O, Only-child; EG, Ethnic group; M, Medical students; D, Dating relationship; R, Religious belief; L, Left-behind experiences on childhood; EF, Educational level of father; EM, Educational level of mother; S, Smoking.

### Overall prevalence of depression

**Figure 2:**
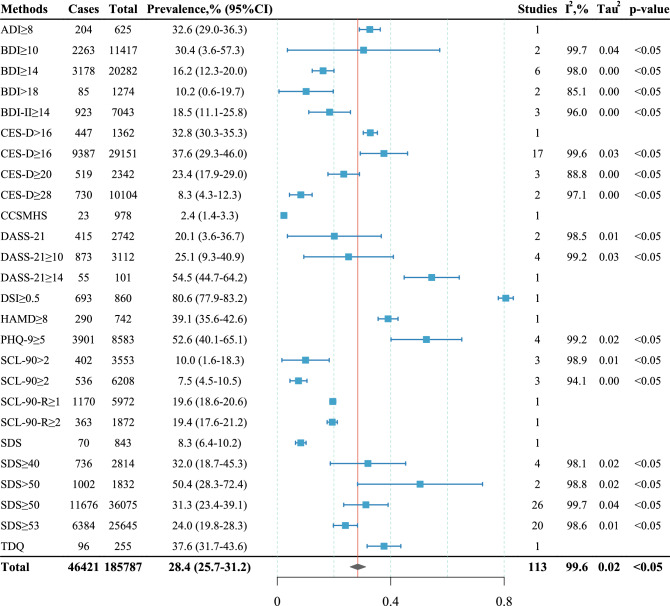
Depression prevalence categorized by different screening methods and cutoff scores.

**Figure 3:**
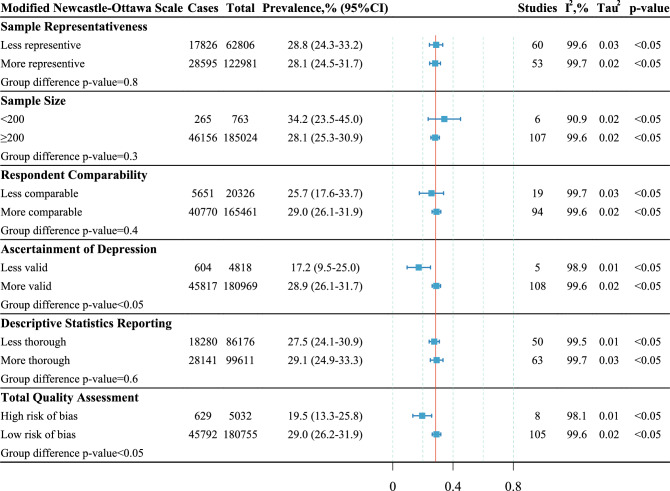
Subgroup analysis of the modified Newcastle–Ottawa Scale.

The overall prevalence of depression among Chinese university students of all the included studies was shown to be 28.4% (n = 185,787), with 95%CI from 25.7% to 31.2% and total heterogeneity I^2^ of 99.6% in a random-effects model (forest plot shown in Appendix-Fig. 1–3). Sensitivity analysis showed that the overall prevalence was affected by less than 0.4% by any individual study, as shown in online Appendix-Table 2 for detail. Figure 2 shows the depression prevalence categorized by different screening methods and cutoff scores. The most commonly used screening method and cutoff score was SDS ≥ 50, which had a prevalence of 31.3% (n = 36,075, 95%CI: 23.4%-39.1%). The second most commonly used screening method and cutoff score was SDS ≥ 53, which had a prevalence of 24.0% (n = 25,645, 95%CI: 19.8%-28.3%). Excluding the 10 studies using a unique screening method and cutoff score, the meta-analysis of the left 103 studies showed a depression prevalence of 28.0% (n = 172,177, 95%CI: 25.2%-30.8%), which had a similar result compared to the full analysis.

**Table 2:** Influencing factors that may affect the prevalence of depression.

Factors	No. of studies	Subgroup	Cases	Total	Prevalence, % (95% CI)	I^2^ (%)	Tau^2^	p-value	Group difference *p* value
Gender	42	Male	9163	38,047	30.3 (25.7–34.9)	99.4	0.02	< 0.05	0.8
		Female	10,097	44,572	29.5 (25.6–33.4)	99.2	0.02	< 0.05	
Family origin	21	Rural	6509	24,804	33.6 (26.4–40.7)	99.6	0.03	< 0.05	0.8
		Urban	5122	21,273	32.2 (25.0–39.4)	99.6	0.03	< 0.05	
Academic grade	18	Non-freshmen	6015	24,851	29.0 (22.9–35.1)	99.4	0.02	< 0.05	0.3
		Freshmen	3418	18,793	25.1 (20.2–30.1)	98.8	0.01	< 0.05	
Only-child	8	Not an only-child	2216	7107	31.1 (19.6–42.7)	99.2	0.03	< 0.05	0.9
		Only-child	1715	5258	29.9 (19.5–40.3)	98.7	0.02	< 0.05	
Ethnic group	7	Others	433	1637	38.4 (25.1–51.7)	97.2	0.03	< 0.05	0.7
		Han	5100	19,374	35.1 (22.1–48.0)	99.8	0.03	< 0.05	
Medical students	6	Medical	1746	8888	25.7 (17.9–33.6)	98.8	0.01	< 0.05	0.5
		Non-medical	1874	12,051	22.7 (16.7–28.6)	98.6	0.01	< 0.05	
Dating relationship	5	Without a bf/gf	1207	3478	36.3 (30.1–42.5)	92.8	0.00	< 0.05	0.3
		Has a bf/gf	488	1483	32.0 (27.9–36.1)	63.8	0.00	< 0.05	
Religious belief	4	Religious	164	379	48.1 (27.6–68.6)	94.2	0.04	< 0.05	0.5
		Irreligious	1294	3242	37.8 (16.8–58.7)	99.4	0.05	< 0.05	
Left-behind experiences on childhood	4	Experienced	551	2005	30.7 (16.5–44.9)	98.1	0.02	< 0.05	0.2
		Non-experienced	2240	9176	20.7 (11.4–30.0)	99	0.01	< 0.05	
Educational level of father	4	≤ 9 years	2138	8695	23.8 (16.8–30.9)	98.1	0.01	< 0.05	0.3
		> 9 years	1456	8038	18.6 (12.4–24.8)	97.7	0.00	< 0.05	
Educational level of mother	3	≤ 9 years	1428	5643	23.0 (10.3–35.7)	99.2	0.01	< 0.05	0.7
		> 9 years	432	2127	19.5 (10.1–28.8)	96.3	0.01	< 0.05	
Smoking	3	Smoker	95	249	32.2 (17.9–46.5)	63.8	0.01	0.1	1.0
		Non-smoker	659	2063	32.3 (28.8–35.8)	60.7	0.00	0.1

Figure 3 shows the subgroup analysis of the modified Newcastle–Ottawa Scale. Students in the studies with low risk of bias had a higher depression prevalence (n = 180,755, 29.0%, 95%CI: 26.2% to 31.9%) than those with high risk of bias (n = 5,032, 19.5%, 95%CI: 13.3% to 25.8%). In the category of ascertainment of depression, a higher prevalence was found in the more valid subgroup than the less valid subgroup. While there was no statistically significant difference in the prevalence estimate of any other category in the modified Newcastle–Ottawa Scale.

**Figure 4:**
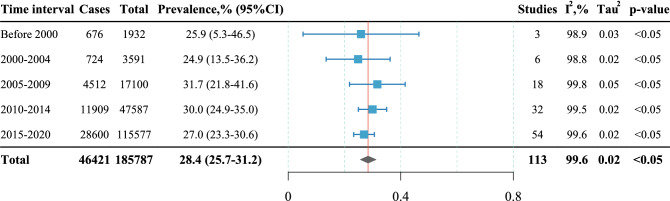
Subgroup analysis of 5-year interval.

Figure 4 shows the depression prevalence variation across time under the subgroup analysis of 5-year interval. Since there were only three studies published before 2000 (earliest in 1992), they were categorized as one subgroup. The depression prevalence was higher in the subgroup of 2005–2009 (n = 17,100, 31.7%, 95%CI: 21.8% to 41.6%) than any other subgroup, however, a *p* value of 0.8 for subgroup differences indicated that there was no statistically significant difference.

### Influencing factors analysis

As reported in the included studies, many factors may affect the prevalence of depression. We pooled and analyzed the data if more than three studies reported the same influencing factor. In total, there were 12 influencing factors, as shown in Table 2. However, the group difference was not statistically significant for any influencing factor. Forest plot of the individual studies of the 12 influencing factors was provided in the online Appendix-Figs. 4–15.

## Discussion

China is now one of the world’s fastest-growing economies^23^. The life quality and mental state of people in China have changed a lot with economic development^24^. Furthermore, many policies were also changed in recent years in China. For example, the one-child policy has been abolished by the Chinese government^25^, China is still expanding enrollment at universities^26^. All these changes not only reflect a dramatic change in the social environment, but also may have an impact on the psychological characteristics of university students in China. Therefore, reliably estimating the prevalence of depression is an important step to making efforts to provide better mental healthcare to university students in the changing environment in China.

In this study, a total of 113 studies were included to estimate the prevalence of depression among Chinese university students. It was shown that the overall depression prevalence was 28.4% (n = 185,787, 95%CI: 25.7% to 31.2%). The overall depression prevalence was relatively high, while it was close to the prevalence estimates in other meta-analyses, such as 30.6% in global university students^1^, 23.8% in Chinese university students^14^, 27.2% in medical students^13^. The overall depression prevalence analysis and subgroup analyses were highly heterogeneous. A significant reason for the heterogeneity is that different categories of method and cutoff score were used in the included studies to screen depression. In addition, many factors, such as population distribution, gender ratio, sample size, also contributed to the heterogeneity.

The screening method and cutoff score have a huge impact on the prevalence of depression. In this study, the estimate of prevalence ranged from 2.4 to 80.6% in different subgroups of the screening methods. Some studies used the same screening method, but different cutoff scores. Usually, a higher cutoff score was accompanied by a lower prevalence of depression. For example, the prevalence of CES-D ≥ 28 (8.3%) was lower than CES-D ≥ 20 (23.4%), and CES-D ≥ 16 (37.6%); the prevalence of SDS ≥ 53 (24.0%) was lower than SDS ≥ 50 (31.3%), and SDS ≥ 40 (32.0%). This is a primary factor that contributes to the heterogeneity of the included studies.

In the summary of influencing factors, there was no statistically significant difference for any group. However, the mean estimate of subgroup prevalence in some influencing factors may be different, such as academic grade (Non-freshmen 29.0%, Freshmen 25.1%).

The pooled data showed that freshmen were less likely to suffer depression compared with non-freshmen, however, this result is contrary to some other studies^27,28^. It is assumed that the management of students played a role in this phenomenon. In China, freshmen in most universities are living in a group. Usually, they go to class together and live in the students’ dormitory with several classmates. After class, many activities have been organized to make them adapt to campus life quickly. They received a lot of attention from their supervisors. In comparison, the students’ lives become free and unconstrained when they enter into a higher grade. They do not need to live together with their classmates and fewer supports will be provided by the supervisors, as the supervisors always think that one year’s campus life is enough for the students’ adaption. However, the students are still facing many challenges, such as academic stress, internship, employment, or interpersonal relationship. All these challenges are stressful to the students, and they have to deal with all the stresses by themselves. Then, the non-freshmen have a higher chance to suffer depression.

The influencing factors analysis also showed that students in the ethnic group of Han or have no religious beliefs are less likely to suffer depression. In Chinese universities, these students are the majority of the population. In the pooled data, the population of the Han ethnic group (n = 19,374) was more than 10 times as that of the minorities (n = 1,637). Similarly, the population which has no religious belief (n = 3,242) is also significantly higher than that has a religious belief (n = 379). Many studies support the evidence that minorities in a population are more likely to suffer depression^29,30^. Maybe more efforts need to be done to support the minority students in the universities.

The educational level of father or mother has an influence on the depression prevalence of Chinese university students. In the factor of father’s educational level, the prevalence was 23.8% (≤ 9 years, n = 8,695, 95%CI: 16.8% to 30.9%) in comparison with 18.6% (> 9 years, n = 8,038, 95%CI: 12.1% to 25.1%). While in the mother’s educational level, the prevalence was 23.0% (≤ 9 years, n = 5,643, 95%CI: 12.7% to 33.3%) in comparison with 19.5% (> 9 years, n = 2127, 95%CI: 11.7% to 27.2%). Students are less likely to suffer depression when their father or mother has a higher educational level. It was also suggested by some studies that father or mother’s education was correlated to the student’s depression^31^. In a family where parents were well educated, students may have more access to financial or psychological support, which may be good for their mental health. However, there are a limited number of studies reported the educational level of parents, maybe more studies need to be done to confirm this hypothesis.

Left-behind experience in childhood indicates that the students were left in rural hometowns while father, or mother, or both, moving to work in cities for a long period during their childhood. It is a negative life event in one’s life^32^. Students with left-behind experiences in childhood are more likely to suffer depression (30.7%) compared with non-experiences (20.7%). This negative life event in childhood may have a relatively long period of effect on the student’s mental health.

There are several limitations in this systematic review and meta-analysis. First, high heterogeneity was found in this meta-analysis. The main reason was that the screening method and cutoff score varied across studies. In addition, the investigated population was not the same across studies. Second, more than half of the included studies were assessed as less representative in sample representativeness, as the investigated population was only from a single major, a single grade, or a single university. Bias may be introduced in the estimate of depression prevalence. Third, the quality of descriptive statistics reporting was low in many studies, much information about the students was not reported. Therefore, in the future, studies estimating depression prevalence among Chinese university students should consider a prospective, multi-center design using a single commonly used screening method and cutoff score to assess depression in random samples of the population.

## Methods

### Database and search strategies

Four electronic databases were searched in this study: Web of Science, PubMed, Chinese National Knowledge Infrastructure, and Wan-fang Database. Search terms were: (depression OR depressive symptom OR depressive disorder OR depressive neurosis OR melancholia) AND (China OR Chinese) AND (college student OR university student OR undergraduate student) AND (observation OR survey OR prevalence OR incidence OR epidemiology OR questionnaire). The language was limited to Chinese and English, and the final search was conducted on May 12, 2020. The detailed database search strategies are supplied in online Appendix-Method 1. Additional eligible studies were identified by manual searching relevant reviews.

### Eligibility criteria

Inclusion criteria were set as follows: (1) the study was conducted among Chinese university students; (2) the study was published in a peer-reviewed journal; (3) a validated method was used to screen depression; (4) the study was published in English or Chinese; (5) the study should include depression as a main or secondary outcome; (6) detailed data of the depression, such as the number of cases of depression, was published to calculate the depression prevalence.

The validated method to screen depression includes various instruments, such as Self-Rating Depression Scale (SDS), Beck Depression Inventory (BDI), Center for Epidemiologic Studies Depression Scale (CESD-S), Patient Health Questionnaire-9 (PHQ-9), and the Symptom Checklist (SCL-90). Some instruments, which may not be commonly used, were also adopted if detailed information about the instrument in the published paper was provided.

Studies were excluded if they met the exclusion criteria: (1) lack of data to calculate the depression prevalence; (2) include non-Chinese university students in the study population, and cannot distinguish depression data of the Chinese university students; (3) repeated studies or studies using the same population; (4) specific disease was associated with the investigated population; (5) depression was measured during or after a major event such as an earthquake, or a disease epidemic.

### Data extraction and quality assessment

Two reviewers (Gao and Xie) independently extracted the data and assessed the qualities of the screened studies. Any disagreement was resolved by discussions with the third reviewer (Wang). For the included studies, data were collected: first author, year of publication, number of depressed students, sample sizes, language, age of the population, male proportion, method to screen depression, all the factors that may affect the prevalence of depression, and geographic location. A modified Newcastle–Ottawa Scale (shown in online Appendix-Method 2) was used to assess the qualities of included studies^13^. The total scores range from 0 to 5, and studies were judged to be at low risk of bias (≥ 3 points) or high risk of bias (< 3 points).

### Statistical analysis

Two reviewers (Gao and Xie) independently performed the data analyses with R Foundation for Statistical Computing (Version 3.6.2). The package of “meta” was used for the calculation of an overall proportion, and the package of “forestplot” was used for the plot of all the figures in this study. The function of “metaprop” was used to estimate the summary effect in the random-effects model with 95% confidence intervals (CI). The I^2^ statistic was used to assess between-study heterogeneity. Sensitivity analysis was conducted to check the stability of the summary result with a series of meta-analyses omitting one study at one time. A stratified meta-analysis was conducted to compare different screening methods, different Newcastle–Ottawa Scale components, and different time intervals. Subgroup analysis was conducted to analyze the influencing factors on the depression prevalence. The analysis was considered as statistically significant only when the p-value was lower than 0.05. Any conflict in the data analysis was discussed by all the reviewers.

## Conclusion

In conclusion, this meta-analysis included 113 studies that reported depression among Chinese university students. The overall depression prevalence was estimated to be 28.4% (n = 185,787, 95%CI: 25.7% to 31.2%). As this prevalence is still high, more efforts need to be done to provide better mental healthcare to university students in China.

## Supplementary information

Supplementary Information.
