# Supplementary material for: Prevalence of depression among Chinese university students: a systematic review and meta-analysis
Source: Sci Rep. 2020 Sep 28;10:15897. doi: 10.1038/s41598-020-72998-1 (PMC7522998; doi:10.1038/s41598-020-72998-1)
Supplement: Supplementary file 1 — Supplementary Information. [file 41598_2020_72998_MOESM1_ESM.pdf]

Prevalence of depression among Chinese university students: A systematic review and meta-analysis

Li Gao [1, 2, *], Yuanchen Xie [3], Chunhua Jia [1], Wei Wang [1, *]

[1] Beijing University of Chinese Medicine, Beijing 100029, China

[2] St Michael's Hospital, University of Toronto, Toronto M5B 1W8, Canada

[3] Zhengzhou University, Zhengzhou 450052, China

* Corresponding author 1: Li Gao, E-mail: gaol@bucm.edu.cn;

* Corresponding author 2: Wei Wang, E-mail: wangwei@bucm.edu.cn;

Appendix

Appendix-Method 1 Database search strategies in detail 3

Appendix-Method 2 Modified Newcastle-Ottawa risk of bias scoring guide 5

Appendix-Table 1 Details of the qualities of the studies assessed by the modified Newcastle-Ottawa scale 7

Appendix-Table 2 Sensitivity analysis of the depression prevalence among Chinese university students 10

Appendix-Fig. 1 Forest plot of the 113 studies (Studies of 1-40) 13

Appendix-Fig. 2 Forest plot of the 113 studies (Studies of 41-80) 14

Appendix-Fig. 3 Forest plot of the 113 studies (Studies of 81-113) 15

Appendix-Fig. 4 Subgroup analysis of gender 16

Appendix-Fig. 5 Subgroup analysis of family origin 17

Appendix-Fig. 6 Subgroup analysis of academic grade 18

Appendix-Fig. 7 Subgroup analysis of only-child or not 19

Appendix-Fig. 8 Subgroup analysis of ethnic group 20

Appendix-Fig. 9 Subgroup analysis of medical students or not 21

Appendix-Fig. 10 Subgroup analysis of dating relationship 22

Appendix-Fig. 11 Subgroup analysis of religious belief 23

Appendix-Fig. 12 Subgroup analysis of left-behind experiences on childhood.......... 24

Appendix-Fig. 13 Subgroup analysis of educational level of father............................. 25

Appendix-Fig. 14 Subgroup analysis of educational level of mother 26

Appendix-Fig. 15 Subgroup analysis of smoking or not.. 27

References 28

Appendix-Method 1 Database search strategies in detail

Web of Science

(depression OR depressive symptom OR depressive disorder OR depressive neurosis OR melancholia)

AND (China OR Chinese)

AND (college student OR university student OR undergraduate student)

AND (observation OR survey OR prevalence OR incidence OR epidemiology OR questionnaire)

(Searched in topic)

PubMed

((((("depression"[Title/Abstract] OR "depressive symptom"[Title/Abstract]) OR "depressive disorder"[Title/Abstract]) OR "depressive neurosis"[Title/Abstract]) OR (((("depressive disorder"[MeSH Terms] OR ("depressive"[All Fields] AND "disorder"[All Fields])) OR "depressive disorder"[All Fields]) OR "melancholia"[All Fields]) OR "melancholias"[All Fields])) AND ("China"[Title/Abstract] OR (((("asian continental ancestry group"[MeSH Terms] OR ((("asian"[All Fields] AND "continental"[All Fields]) AND "ancestry"[All Fields]) AND "group"[All Fields])) OR "asian continental ancestry group"[All Fields]) OR "chinese"[All Fields]) OR "chineses"[All Fields]))) AND (("college student*"[Title/Abstract] OR "university student*"[Title/Abstract]) OR (((("undergraduate"[All Fields] OR "undergraduate s"[All Fields]) OR "undergraduated"[All Fields]) OR "undergraduates"[All Fields]) AND "student*"[All Fields]))) AND ((((("observation"[Title/Abstract] OR "survey"[Title/Abstract]) OR "prevalence"[Title/Abstract]) OR "incidence"[Title/Abstract]) OR "epidemiology"[Title/Abstract]) OR (((((("questionnair"[All Fields] OR "questionnaires"[All Fields]) OR "surveys and questionnaires"[MeSH Terms]) OR ("surveys"[All Fields] AND "questionnaires"[All Fields])) OR "surveys and questionnaires"[All Fields]) OR "questionnaire"[All Fields]) OR "questionnaires"[All Fields]))

Chinese National Knowledge Infrastructure (a Chinese database)

(抑郁 OR 抑郁症 OR 抑郁性神经症 OR 忧郁症) AND (大学生 OR 高校学生 OR 本科生) AND (调查问卷 OR 发病率 OR 流行病学 OR 横断面研究)

(Searched in Topic)

Wan-fang Database (a Chinese database)

(抑郁 OR 抑郁症 OR 抑郁性神经症 OR 忧郁症) AND (大学生 OR 高校学生 OR 本科生) AND (调查问卷 OR 发病率 OR 流行病学 OR 横断面研究)

(Searched in Topic)

Appendix-Method 2 Modified Newcastle-Ottawa risk of bias scoring guide

(1) Sample representativeness:

1 point: Population contained students of multiple majors and grades in multiple universities.

0 points: Population contained students only in a single major, a single grade, or a single university.

(2) Sample size:

1 point: Sample size was greater than or equal to 200 participants.

0 points: Sample size was less than 200 participants.

(3) Non-respondents:

1 point: Comparability between respondent and non-respondent characteristics was established with a satisfactory response rate.

0 points: The comparability between respondents and non-respondents was unsatisfactory, the response rate was unsatisfactory, or there was no description of the response rate or the characteristics of the responders or non-responders.

(4) Ascertainment of depression or depressive symptoms:

1 point: The study employed a commonly used measurement tool with a valid cutoff score (e.g., BDI ≥10, BDI-II ≥14, CES-D ≥16, PHQ-9 ≥10, Zung-SDS ≥50).

0 points: The study employed an infrequently used measurement tool, a commonly used measurement tool with an invalid cutoff score, or any tool with published sensitivity/specificity values <70% (e.g., the PRIME-MD screening instrument).

(5) Quality of descriptive statistics reporting:

1 point: The study reported descriptive statistics to describe the population (e.g., age, sex, class year) with proper measures of dispersion (e.g., mean, standard deviation).

0 points: The study did not report descriptive statistics, incompletely reported descriptive statistics, or did not report measures of dispersion.

Legend: The individual components listed above are summed to generate a total modified Newcastle-Ottawa risk of bias score for each study. Total scores range from 0 to 5. For the total score grouping, studies were judged to be of low risk of bias (≥3

points) or high risk of bias (<3 points).

Appendix-Table 1 Details of the qualities of the studies assessed by the modified Newcastle-Ottawa scale

Study ID	Sample representativeness	Sample size	Non-respondents	Depression ascertainment	Descriptive statistics reporting	**Total score**
Cai (2017) [1]	1	1	1	1	1	**5**
Chai (2011) [2]	0	1	1	1	0	**3**
Chan (1992) [3]	0	0	0	1	1	**2**
Chang (2011) [4]	0	1	0	0	0	**1**
Chen (2013) [5]	1	1	1	1	1	**5**
Chen (2015) [6]	0	1	1	1	0	**3**
Chen (2016) [7]	1	1	1	1	1	**5**
Cheung (2016) [8]	0	1	1	1	1	**4**
Chou (2018) [9]	1	1	1	1	0	**4**
Deng (2011) [10]	1	1	1	1	1	**5**
Dong (2019) [11]	1	1	1	1	1	**5**
Du (1999) [12]	0	1	0	1	1	**3**
Fang (2017) [13]	1	1	1	1	0	**4**
Feng (2005) [14]	0	1	1	1	1	**4**
Feng (2014) [15]	0	1	1	1	1	**4**
Fu (2010) [16]	1	1	1	1	0	**4**
Gao (2008) [17]	0	1	1	1	1	**4**
Gao (2018) [18]	0	1	1	1	1	**4**
Guo (2013) [19]	1	1	1	1	1	**5**
Guo (2019) [20]	0	1	0	1	1	**3**
Guo (2020) [21]	0	1	1	1	1	**4**
Hall (2018) [22]	0	0	1	1	1	**3**
Han (2011) [23]	0	1	1	1	0	**3**
Han (2015) [24]	0	1	0	0	0	**1**
Han (2018) [25]	1	1	1	1	0	**4**
He (2015) [26]	1	1	1	1	1	**5**
Hou (2014) [27]	0	1	0	0	0	**1**
Hou H (2018) [28]	1	1	1	1	1	**5**
Hou Y (2018) [29]	0	1	1	1	0	**3**
Hu (2012) [30]	1	1	1	1	0	**4**
Hua (2008) [31]	0	0	1	1	0	**2**
Jiang (2011) [32]	1	1	1	1	0	**4**
Jin (2014) [33]	1	1	1	1	0	**4**
Kang (2017) [34]	1	1	1	1	0	**4**
Lei (2018) [35]	1	1	1	1	0	**4**
Li (2018) [36]	1	1	1	1	0	**4**
Lin (2018) [37]	0	1	1	1	0	**3**
Liu (1997) [38]	0	1	0	1	1	**3**

Liu (2011) [39]	0	0	1	1	1	**3**
Liu (2014) [40]	0	1	1	1	1	**4**
Liu (2015) [41]	0	1	1	1	0	**3**
Liu (2017) [42]	1	1	1	1	1	**5**
Liu (2019) [43]	1	1	1	1	0	**4**
Liu (2020) [44]	1	1	1	1	0	**4**
Lu (2015) [45]	0	1	0	1	1	**3**
Luo (2004) [46]	0	1	1	1	1	**4**
Ma (2019) [47]	0	1	1	1	1	**4**
Niu (2010) [48]	0	1	1	1	0	**3**
Pan (2016) [49]	1	1	1	1	0	**4**
Peng (2010) [50]	0	1	1	1	0	**3**
Shao (2020) [51]	0	1	0	1	1	**3**
Shen (2016) [52]	1	1	1	1	1	**5**
Shi (2016) [53]	1	1	1	1	1	**5**
Sobowale (2014) [54]	1	1	1	1	0	**4**
Song (2008) [55]	1	1	1	1	1	**5**
Sun (2008) [56]	0	1	1	1	1	**4**
Sun (2011) [57]	1	1	1	1	0	**4**
Sun (2013) [58]	0	1	1	1	0	**3**
Sun (2017) [59]	1	1	1	1	0	**4**
Tan (2012) [60]	0	1	1	1	0	**3**
Tang F (2018) [61]	1	1	1	1	0	**4**
Tang W (2018) [62]	1	1	1	1	1	**5**
Tong (2016) [63]	1	1	0	1	0	**3**
Wang (2007) [64]	0	1	1	1	0	**3**
Wang (2011) [65]	1	1	0	1	0	**3**
Wang (2012) [66]	0	1	0	1	0	**2**
Wang (2013) [67]	0	1	1	1	1	**4**
Wang L (2019) [68]	1	1	0	0	0	**2**
Wang M (2019) [69]	1	1	1	1	1	**5**
Wang Z (2019) [70]	0	1	1	1	0	**3**
Wei (2011) [71]	1	1	1	1	0	**4**
Wu (2007) [72]	1	1	1	1	0	**4**
Wu (2015) [73]	1	1	1	1	1	**5**
Wu (2016) [74]	0	1	1	1	1	**4**
Xi (2010) [75]	0	1	1	1	0	**3**
Xiao (2006) [76]	1	1	1	1	1	**5**
Xiao (2016) [77]	1	1	1	1	0	**4**
Xu (2002) [78]	0	1	1	1	1	**4**
Xu (2003) [79]	0	1	0	1	1	**3**
Xu (2014) [80]	1	1	1	1	1	**5**
Xu (2016) [81]	1	1	1	1	1	**5**
Xu (2020) [82]	1	1	1	1	1	**5**

Yang (2008) [83]	0	1	1	1	0	**3**
Yang C (2013) [84]	0	1	1	1	1	**4**
Yang H (2013) [85]	0	1	1	1	0	**3**
Yang M (2007) [86]	0	0	1	1	0	**2**
Yang X (2007) [87]	0	1	1	1	1	**4**
Ye (2016) [88]	1	1	0	1	1	**4**
Yen (2011) [89]	1	1	1	1	1	**5**
Yu (2011) [90]	0	1	1	1	0	**3**
Yu (2015) [91]	1	1	1	1	0	**4**
Zeng (2003) [92]	0	1	1	1	1	**4**
Zeng (2006) [93]	0	1	1	1	0	**3**
Zeng (2019) [94]	1	1	1	0	1	**4**
Zhai (2005) [95]	0	1	1	1	1	**4**
Zhang (2004) [96]	1	1	0	1	1	**4**
Zhang (2005) [97]	0	1	0	1	1	**3**
Zhang (2006) [98]	1	1	1	1	1	**5**
Zhang (2015) [99]	1	1	1	1	1	**5**
Zhang (2018) [100]	0	1	0	1	1	**3**
Zhang (2020) [101]	0	1	1	1	1	**4**
Zhao (2018) [102]	1	1	1	1	1	**5**
Zheng (2008) [103]	0	1	1	1	1	**4**
Zheng (2016) [104]	0	1	1	1	1	**4**
Zhong (2011) [105]	0	1	1	1	0	**3**
Zhou (2003) [106]	0	0	1	1	1	**3**
Zhou (2009) [107]	0	1	1	1	1	**4**
Zhou (2018) [108]	1	1	1	1	0	**4**
Zhu (2019) [109]	0	1	1	1	1	**4**
Zong (2010) [110]	1	1	1	1	0	**4**
Zou (2007) [111]	0	1	1	1	1	**4**
Zou & Sun (2018) [112]	1	1	0	1	1	**4**
Zou & Wang (2018) [113]	0	1	1	1	1	**4**

Appendix-Table 2 Sensitivity analysis of the depression prevalence among Chinese university students

Study Omitted	Prevalence (%)	LCI (%)	UCI (%)	τ^2	I^2 (%)
Cai (2017) [1]	28.4	25.7	31.1	0.0215	99.6
Chai (2011) [2]	28.5	25.7	31.2	0.0216	99.6
Chan (1992) [3]	28.5	25.8	31.3	0.0215	99.6
Chang (2011) [4]	28.3	25.6	31.1	0.0215	99.6
Chen (2013) [5]	28.6	25.8	31.4	0.0221	99.6
Chen (2015) [6]	28.4	25.7	31.1	0.0215	99.6
Chen (2016) [7]	28.4	25.7	31.2	0.0215	99.6
Cheung (2016) [8]	28.4	25.6	31.1	0.0215	99.6
Chou (2018) [9]	28.4	25.7	31.2	0.0215	99.6
Deng (2011) [10]	28.5	25.7	31.2	0.0217	99.6
Dong (2019) [11]	28.4	25.7	31.1	0.0215	99.6
Du (1999) [12]	28.3	25.6	31.0	0.0213	99.6
Fang (2017) [13]	28.1	25.5	30.8	0.0208	99.6
Feng (2005) [14]	28.5	25.8	31.3	0.0215	99.6
Feng (2014) [15]	28.6	25.8	31.3	0.0216	99.6
Fu (2010) [16]	28.2	25.5	31.0	0.0213	99.6
Gao (2008) [17]	28.4	25.7	31.1	0.0215	99.6
Gao (2018) [18]	28.5	25.8	31.3	0.0216	99.6
Guo (2013) [19]	28.3	25.6	31.0	0.0214	99.6
Guo (2019) [20]	28.3	25.6	31.0	0.0214	99.6
Guo (2020) [21]	28.5	25.7	31.2	0.0218	99.6
Hall (2018) [22]	28.2	25.5	30.9	0.0214	99.6
Han (2011) [23]	28.5	25.8	31.2	0.0215	99.6
Han (2015) [24]	28.6	25.9	31.4	0.0216	99.6
Han (2018) [25]	28.4	25.6	31.1	0.0215	99.6
He (2015) [26]	28.4	25.6	31.1	0.0215	99.6
Hou (2014) [27]	28.7	25.9	31.4	0.0213	99.6
Hou H (2018) [28]	28.6	25.8	31.4	0.0220	99.6
Hou Y (2018) [29]	28.6	25.8	31.3	0.0218	99.6
Hu (2012) [30]	28.4	25.7	31.1	0.0215	99.6
Hua (2008) [31]	28.5	25.7	31.2	0.0215	99.6
Jiang (2011) [32]	28.4	25.7	31.1	0.0215	99.6
Jin (2014) [33]	28.4	25.6	31.1	0.0214	99.6
Kang (2017) [34]	28.5	25.8	31.2	0.0215	99.6
Lei (2018) [35]	28.6	25.9	31.4	0.0220	99.6
Li (2018) [36]	28.4	25.7	31.1	0.0215	99.6
Lin (2018) [37]	28.6	25.8	31.3	0.0216	99.6
Liu (1997) [38]	28.5	25.8	31.3	0.0215	99.6
Liu (2011) [39]	28.4	25.7	31.2	0.0215	99.6
Liu (2014) [40]	28.3	25.6	31.1	0.0214	99.6

Liu (2015) [41]	28.5	25.8	31.3	0.0216	99.6
Liu (2017) [42]	28.5	25.8	31.2	0.0216	99.6
Liu (2019) [43]	28.4	25.6	31.1	0.0214	99.6
Liu (2020) [44]	28.5	25.8	31.3	0.0216	99.6
Lu (2015) [45]	28.1	25.4	30.8	0.0208	99.6
Luo (2004) [46]	28.2	25.5	30.9	0.0214	99.6
Ma (2019) [47]	28.5	25.8	31.3	0.0216	99.6
Niu (2010) [48]	28.5	25.8	31.2	0.0215	99.6
Pan (2016) [49]	28.5	25.7	31.3	0.0223	99.6
Peng (2010) [50]	28.2	25.5	30.9	0.0211	99.6
Shao (2020) [51]	28.2	25.5	30.8	0.0207	99.6
Shen (2016) [52]	28.4	25.7	31.1	0.0215	99.6
Shi (2016) [53]	28.1	25.5	30.7	0.0194	99.6
Sobowale (2014) [54]	28.1	25.4	30.8	0.0213	99.6
Song (2008) [55]	28.4	25.7	31.1	0.0215	99.6
Sun (2008) [56]	28.6	25.9	31.4	0.0215	99.6
Sun (2011) [57]	28.5	25.7	31.3	0.0226	99.6
Sun (2013) [58]	28.0	25.3	30.7	0.0207	99.6
Sun (2017) [59]	28.6	25.8	31.4	0.0222	99.6
Tan (2012) [60]	28.5	25.8	31.3	0.0215	99.6
Tang F (2018) [61]	28.5	25.7	31.3	0.0221	99.6
Tang W (2018) [62]	28.4	25.6	31.1	0.0214	99.6
Tong (2016) [63]	28.5	25.8	31.3	0.0217	99.6
Wang (2007) [64]	28.4	25.7	31.2	0.0215	99.6
Wang (2011) [65]	28.6	25.9	31.4	0.0215	99.6
Wang (2012) [66]	28.5	25.8	31.2	0.0215	99.6
Wang (2013) [67]	28.5	25.7	31.2	0.0216	99.6
Wang L (2019) [68]	28.6	25.8	31.3	0.0217	99.6
Wang M (2019) [69]	28.5	25.7	31.2	0.0219	99.6
Wang Z (2019) [70]	28.4	25.6	31.1	0.0215	99.6
Wei (2011) [71]	28.3	25.6	31.1	0.0215	99.6
Wu (2007) [72]	28.5	25.7	31.2	0.0216	99.6
Wu (2015) [73]	28.5	25.8	31.3	0.0220	99.6
Wu (2016) [74]	28.5	25.8	31.3	0.0218	99.6
Xi (2010) [75]	28.5	25.8	31.2	0.0215	99.6
Xiao (2006) [76]	28.3	25.6	31.1	0.0214	99.6
Xiao (2016) [77]	28.5	25.8	31.2	0.0215	99.6
Xu (2002) [78]	28.6	25.8	31.3	0.0215	99.6
Xu (2003) [79]	28.5	25.7	31.2	0.0216	99.6
Xu (2014) [80]	28.5	25.7	31.2	0.0215	99.6
Xu (2016) [81]	28.4	25.7	31.2	0.0215	99.6
Xu (2020) [82]	28.3	25.6	31.0	0.0208	99.6
Yang (2008) [83]	28.3	25.6	31.0	0.0214	99.6
Yang C (2013) [84]	28.2	25.5	30.9	0.0212	99.6

Yang H (2013) [85]	28.3	25.6	31.0	0.0213	99.6
Yang M (2007) [86]	28.3	25.6	31.0	0.0215	99.6
Yang X (2007) [87]	28.7	26.0	31.4	0.0210	99.6
Ye (2016) [88]	28.4	25.6	31.1	0.0215	99.6
Yen (2011) [89]	28.6	25.8	31.3	0.0218	99.6
Yu (2011) [90]	28.5	25.7	31.2	0.0215	99.6
Yu (2015) [91]	28.6	25.8	31.4	0.0220	99.6
Zeng (2003) [92]	28.6	25.8	31.3	0.0215	99.6
Zeng (2006) [93]	28.2	25.5	31.0	0.0214	99.6
Zeng (2019) [94]	28.4	25.7	31.2	0.0215	99.6
Zhai (2005) [95]	28.5	25.7	31.2	0.0215	99.6
Zhang (2004) [96]	28.6	25.9	31.4	0.0215	99.6
Zhang (2005) [97]	28.3	25.6	31.0	0.0213	99.6
Zhang (2006) [98]	28.0	25.3	30.6	0.0200	99.6
Zhang (2015) [99]	28.5	25.7	31.2	0.0216	99.6
Zhang (2018) [100]	28.5	25.7	31.2	0.0215	99.6
Zhang (2020) [101]	28.6	25.9	31.3	0.0215	99.6
Zhao (2018) [102]	28.3	25.5	31.0	0.0214	99.6
Zheng (2008) [103]	28.1	25.4	30.8	0.0208	99.6
Zheng (2016) [104]	28.4	25.7	31.1	0.0215	99.6
Zhong (2011) [105]	28.3	25.6	31.1	0.0214	99.6
Zhou (2003) [106]	28.3	25.6	31.1	0.0215	99.6
Zhou (2009) [107]	28.6	25.9	31.4	0.0216	99.6
Zhou (2018) [108]	28.6	25.9	31.4	0.0216	99.6
Zhu (2019) [109]	28.4	25.6	31.1	0.0211	99.6
Zong (2010) [110]	28.5	25.8	31.2	0.0215	99.6
Zou (2007) [111]	28.5	25.8	31.3	0.0215	99.6
Zou & Sun (2018) [112]	28.6	25.9	31.4	0.0215	99.6
Zou & Wang (2018) [113]	28.6	25.9	31.3	0.0215	99.6

Appendix-Fig. 1 Forest plot of the 113 studies (Studies of 1-40)

Study ID	Cases	Total	Prevalence,% (95%CI)	Weight,%
Cai (2017)	421	1327	31.7 (29.2-34.3)	0.9
Chai (2011)	400	1681	23.8 (21.8-25.9)	0.9
Chan (1992)	15	95	15.8 (9.1-24.7)	0.8
Chang (2011)	96	255	37.6 (31.7-43.9)	0.9
Chen (2013)	617	5245	11.8 (10.9-12.7)	0.9
Chen (2015)	204	625	32.6 (29.0-36.5)	0.9
Chen (2016)	141	501	28.1 (24.2-32.3)	0.9
Cheung (2016)	232	661	35.1 (31.5-38.9)	0.9
Chou (2018)	89	324	27.5 (22.7-32.7)	0.9
Deng (2011)	650	2768	23.5 (21.9-25.1)	0.9
Dong (2019)	447	1362	32.8 (30.3-35.4)	0.9
Du (1999)	564	1277	44.2 (41.4-46.9)	0.9
Fang (2017)	885	1475	60.0 (57.4-62.5)	0.9
Feng (2005)	79	480	16.5 (13.3-20.1)	0.9
Feng (2014)	117	1106	10.6 (8.8-12.5)	0.9
Fu (2010)	321	631	50.9 (46.9-54.8)	0.9
Gao (2008)	79	253	31.2 (25.6-37.3)	0.9
Gao (2018)	126	730	17.3 (14.6-20.2)	0.9
Guo (2013)	329	745	44.2 (40.6-47.8)	0.9
Guo (2019)	139	306	45.4 (39.8-51.2)	0.9
Guo (2020)	697	3278	21.3 (19.9-22.7)	0.9
Hall (2018)	55	101	54.5 (44.2-64.4)	0.8
Han (2011)	80	381	21.0 (17.0-25.4)	0.9
Han (2015)	70	843	8.3 (6.5-10.4)	0.9
Han (2018)	265	788	33.6 (30.3-37.0)	0.9
He (2015)	398	1186	33.6 (30.9-36.3)	0.9
Hou (2014)	23	978	2.4 (1.5-3.5)	0.9
Hou H (2018)	401	4119	9.7 (8.8-10.7)	0.9
Hou Y (2018)	333	2519	13.2 (11.9-14.6)	0.9
Hu (2012)	101	307	32.9 (27.7-38.5)	0.9
Hua (2008)	23	98	23.5 (15.5-33.1)	0.8
Jiang (2011)	1893	6009	31.5 (30.3-32.7)	0.9
Jin (2014)	395	1095	36.1 (33.2-39.0)	0.9
Kang (2017)	102	519	19.7 (16.3-23.3)	0.9
Lei (2018)	495	7842	6.3 (5.8-6.9)	0.9
Li (2018)	203	629	32.3 (28.6-36.1)	0.9
Lin (2018)	120	913	13.1 (11.0-15.5)	0.9
Liu (1997)	97	560	17.3 (14.3-20.7)	0.9
Liu (2011)	54	185	29.2 (22.8-36.3)	0.9
Liu (2014)	312	804	38.8 (35.4-42.3)	0.9

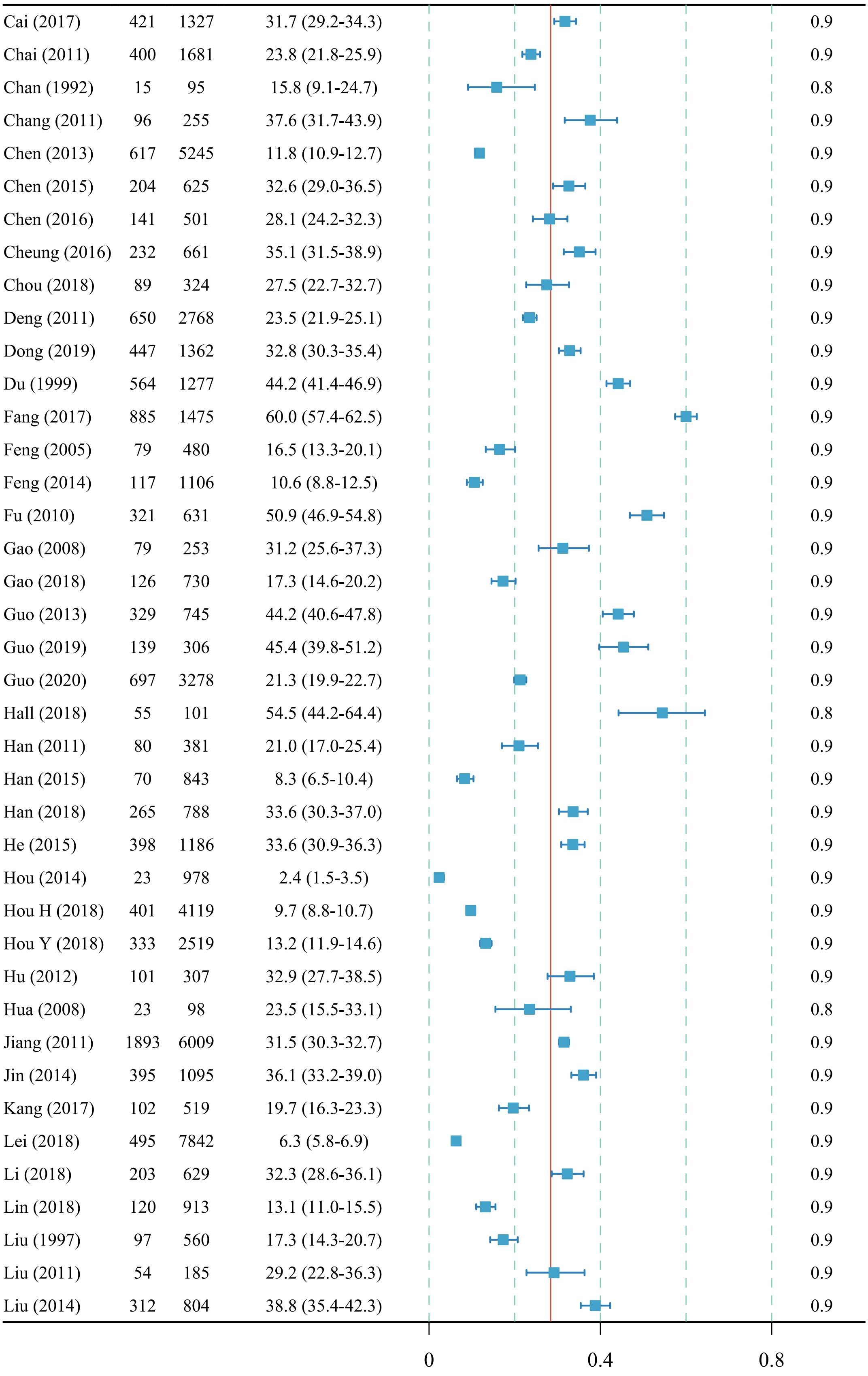

Appendix-Fig. 2 Forest plot of the 113 studies (Studies of 41-80)

Study ID	Cases	Total	Prevalence,% (95%CI)	Weight,%
Liu (2015)	137	755	18.1 (15.5-21.1)	0.9
Liu (2017)	198	1006	19.7 (17.3-22.3)	0.9
Liu (2019)	490	1401	35.0 (32.5-37.5)	0.9
Liu (2020)	300	1505	19.9 (17.9-22.0)	0.9
Lu (2015)	687	1048	65.6 (62.6-68.4)	0.9
Luo (2004)	152	275	55.3 (49.2-61.2)	0.9
Ma (2019)	152	960	15.8 (13.6-18.3)	0.9
Niu (2010)	132	609	21.7 (18.5-25.2)	0.9
Pan (2016)	1751	8819	19.9 (19.0-20.7)	0.9
Peng (2010)	653	1178	55.4 (52.5-58.3)	0.9
Shao (2020)	1183	2057	57.5 (55.3-59.7)	0.9
Shen (2016)	594	1931	30.8 (28.7-32.9)	0.9
Shi (2016)	1954	2925	66.8 (65.1-68.5)	0.9
Sobowale (2014)	226	348	64.9 (59.7-70.0)	0.9
Song (2008)	551	1677	32.9 (30.6-35.2)	0.9
Sun (2008)	53	1171	4.5 (3.4-5.9)	0.9
Sun (2011)	1699	10140	16.8 (16.0-17.5)	0.9
Sun (2013)	510	690	73.9 (70.5-77.2)	0.9
Sun (2017)	708	5989	11.8 (11.0-12.7)	0.9
Tan (2012)	89	588	15.1 (12.3-18.3)	0.9
Tang F (2018)	1170	5972	19.6 (18.6-20.6)	0.9
Tang W (2018)	908	2563	35.4 (33.6-37.3)	0.9
Tong (2016)	363	1872	19.4 (17.6-21.3)	0.9
Wang (2007)	396	1440	27.5 (25.2-29.9)	0.9
Wang (2011)	45	930	4.8 (3.6-6.4)	0.9
Wang (2012)	96	457	21.0 (17.4-25.0)	0.9
Wang (2013)	438	1687	26.0 (23.9-28.1)	0.9
Wang L (2019)	259	2198	11.8 (10.5-13.2)	0.9
Wang M (2019)	1595	6284	25.4 (24.3-26.5)	0.9
Wang Z (2019)	236	667	35.4 (31.8-39.1)	0.9
Wei (2011)	151	391	38.6 (33.8-43.6)	0.9
Wu (2007)	303	1334	22.7 (20.5-25.1)	0.9
Wu (2015)	754	4747	15.9 (14.9-17.0)	0.9
Wu (2016)	392	2521	15.5 (14.2-17.0)	0.9
Xi (2010)	84	402	20.9 (17.0-25.2)	0.9
Xiao (2006)	218	558	39.1 (35.0-43.3)	0.9
Xiao (2016)	105	520	20.2 (16.8-23.9)	0.9
Xu (2002)	31	211	14.7 (10.2-20.2)	0.9
Xu (2003)	381	1750	21.8 (19.9-23.8)	0.9
Xu (2014)	175	763	22.9 (20.0-26.1)	0.9

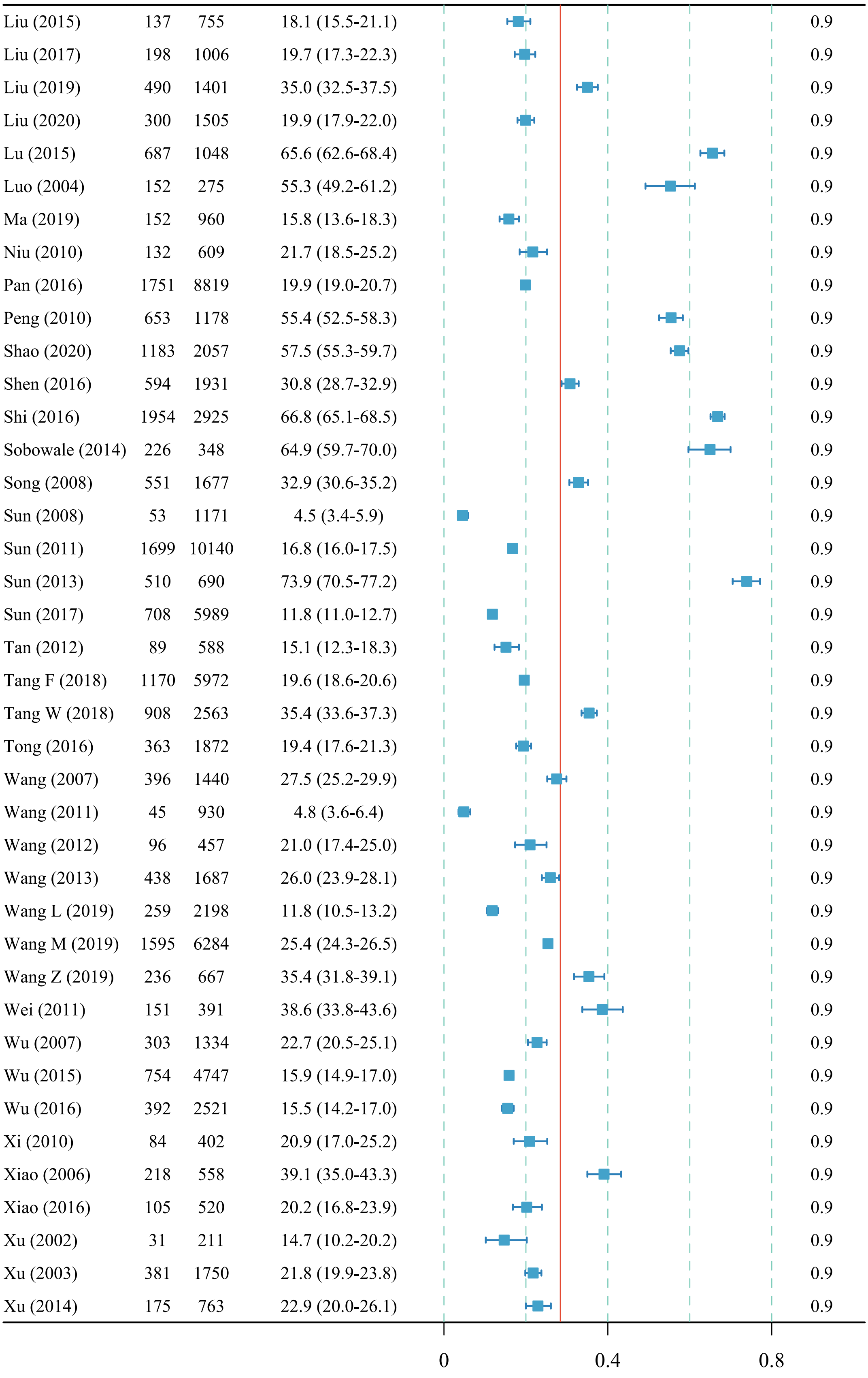

Appendix-Fig. 3 Forest plot of the 113 studies (Studies of 81-113)

Study ID	Cases	Total	Prevalence,% (95%CI)	Weight,%
Xu (2016)	566	1907	29.7 (27.6-31.8)	0.9
Xu (2020)	2080	4624	45.0 (43.5-46.4)	0.9
Yang (2008)	98	222	44.1 (37.5-50.9)	0.9
Yang C (2013)	687	1372	50.1 (47.4-52.8)	0.9
Yang H (2013)	815	1972	41.3 (39.1-43.5)	0.9
Yang M (2007)	47	108	43.5 (34.0-53.4)	0.8
Yang X (2007)	113	3744	3.0 (2.5-3.6)	0.9
Ye (2016)	820	2422	33.9 (32.0-35.8)	0.9
Yen (2011)	235	2262	10.4 (9.2-11.7)	0.9
Yu (2011)	140	600	23.3 (20.0-26.9)	0.9
Yu (2015)	538	4582	11.7 (10.8-12.7)	0.9
Zeng (2003)	40	302	13.2 (9.6-17.6)	0.9
Zeng (2006)	205	408	50.2 (45.3-55.2)	0.9
Zeng (2019)	156	544	28.7 (24.9-32.7)	0.9
Zhai (2005)	114	509	22.4 (18.8-26.3)	0.9
Zhang (2004)	49	877	5.6 (4.2-7.3)	0.9
Zhang (2005)	613	1351	45.4 (42.7-48.1)	0.9
Zhang (2006)	693	860	80.6 (77.8-83.2)	0.9
Zhang (2015)	467	1853	25.2 (23.2-27.2)	0.9
Zhang (2018)	112	468	23.9 (20.1-28.1)	0.9
Zhang (2020)	25	265	9.4 (6.2-13.6)	0.9
Zhao (2018)	136	298	45.6 (39.9-51.5)	0.9
Zheng (2008)	784	1274	61.5 (58.8-64.2)	0.9
Zheng (2016)	101	324	31.2 (26.2-36.5)	0.9
Zhong (2011)	290	742	39.1 (35.6-42.7)	0.9
Zhou (2003)	71	176	40.3 (33.0-48.0)	0.8
Zhou (2009)	70	1179	5.9 (4.7-7.4)	0.9
Zhou (2018)	90	1159	7.8 (6.3-9.5)	0.9
Zhu (2019)	3648	10174	35.9 (34.9-36.8)	0.9
Zong (2010)	56	266	21.1 (16.3-26.5)	0.9
Zou (2007)	73	434	16.8 (13.4-20.7)	0.9
Zou & Sun (2018)	39	582	6.7 (4.8-9.0)	0.9
Zou & Wang (2018)	63	587	10.7 (8.3-13.5)	0.9
**Total**	**46421**	**185787**	**28.4 (25.7-31.2)**	**100.0**

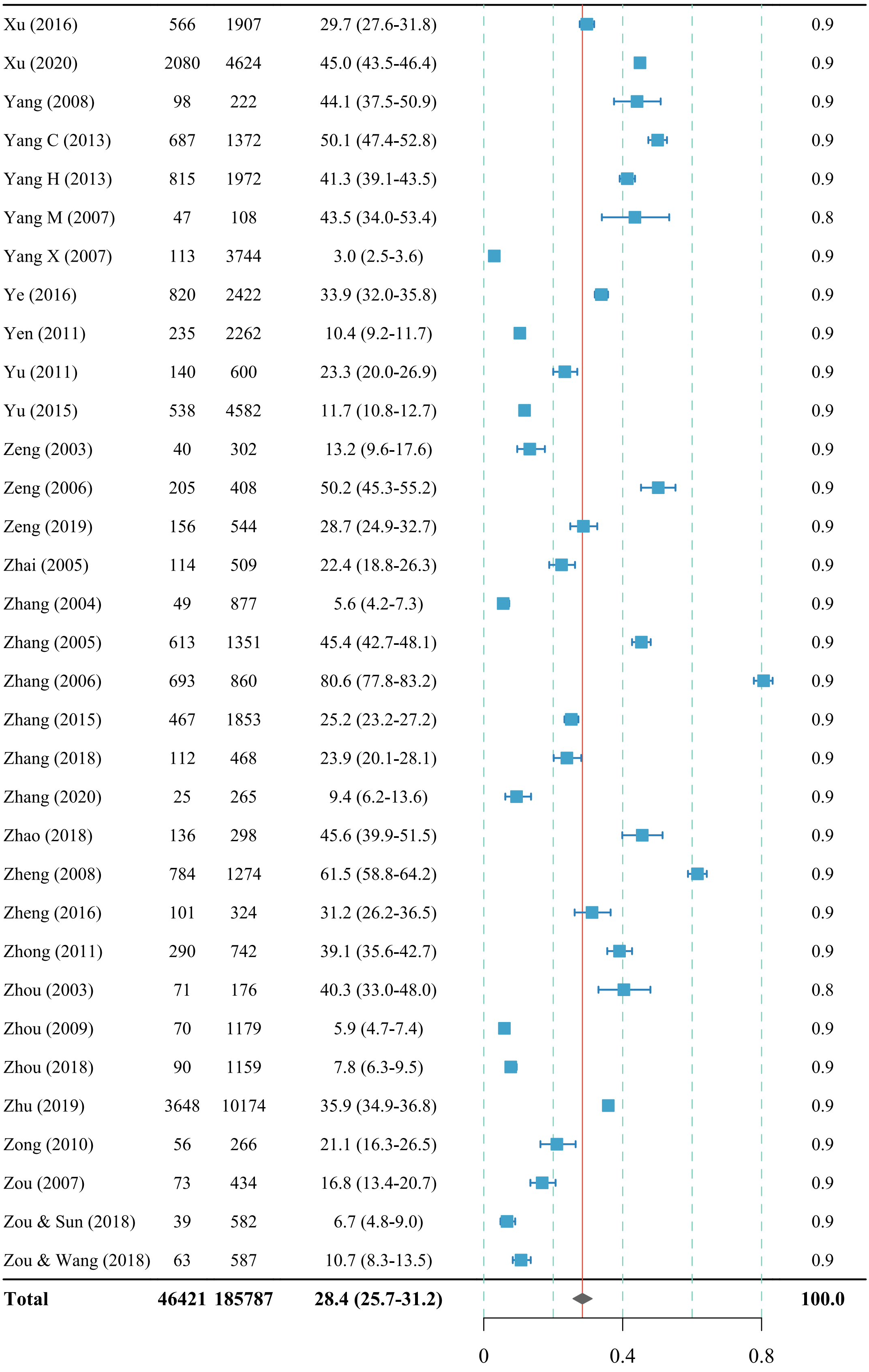

Appendix-Fig. 4 Subgroup analysis of gender

Study ID	Cases	Total	Prevalence,% (95%CI)	Weight,%
Chai (2011)	208	900	23.1 (20.4-26.0)	2.4
Chen (2013)	306	2563	11.9 (10.7-13.3)	2.4
Cheung (2016)	70	182	38.5 (31.4-45.9)	2.3
Guo (2019)	68	151	45.0 (36.9-53.3)	2.3
He (2015)	142	432	32.9 (28.5-37.5)	2.4
Hou H (2018)	76	1114	6.8 (5.4-8.5)	2.4
Hou Y (2018)	134	970	13.8 (11.7-16.1)	2.4
Hu (2012)	48	146	32.9 (25.3-41.1)	2.3
Hua (2008)	13	61	21.3 (11.9-33.7)	2.2
Jiang (2011)	1042	3199	32.6 (30.9-34.2)	2.4
Jin (2014)	177	571	31.0 (27.2-35.0)	2.4
Li (2018)	94	270	34.8 (29.1-40.8)	2.4
Lin (2018)	63	402	15.7 (12.3-19.6)	2.4
Liu (2014)	147	363	40.5 (35.4-45.7)	2.4
Liu (2015)	22	132	16.7 (10.7-24.1)	2.3
Lu (2015)	455	695	65.5 (61.8-69.0)	2.4
Niu (2010)	77	322	23.9 (19.4-29.0)	2.4
Pan (2016)	845	3415	24.7 (23.3-26.2)	2.4
Peng (2010)	418	786	53.2 (49.6-56.7)	2.4
Shen (2016)	292	628	46.5 (42.5-50.5)	2.4
Shao (2020)	339	603	56.2 (52.2-60.2)	2.4
Song (2008)	262	860	30.5 (27.4-33.7)	2.4
Sun (2011)	738	4680	15.8 (14.7-16.8)	2.4
Sun (2013)	414	558	74.2 (70.4-77.8)	2.4
Sun (2017)	325	3196	10.2 (9.1-11.3)	2.4
Tan (2012)	53	294	18.0 (13.8-22.9)	2.4
Tang W (2018)	561	1679	33.4 (31.2-35.7)	2.4
Wang (2013)	193	764	25.3 (22.2-28.5)	2.4
Wu (2015)	320	1973	16.2 (14.6-17.9)	2.4
Xiao (2006)	111	247	44.9 (38.6-51.4)	2.3
Xu (2002)	14	82	17.1 (9.7-27.0)	2.3
Xu (2014)	18	102	17.6 (10.8-26.4)	2.3
Yang H (2013)	268	616	43.5 (39.5-47.5)	2.4
Yang X (2007)	93	2825	3.3 (2.7-4.0)	2.5
Yu (2011)	21	75	28.0 (18.2-39.6)	2.2
Zeng (2006)	113	226	50.0 (43.3-56.7)	2.3
Zhai (2005)	53	209	25.4 (19.6-31.8)	2.4
Zhang (2005)	259	577	44.9 (40.8-49.0)	2.4
Zheng (2016)	39	150	26.0 (19.2-33.8)	2.3
Zhong (2011)	219	509	43.0 (38.7-47.5)	2.4
Zhou (2003)	31	88	35.2 (25.3-46.1)	2.2
Zhou (2009)	22	432	5.1 (3.2-7.6)	2.4
**Male**	**9163**	**38047**	**30.3 (25.7-34.9)**	**100.0**
Heterogeneity: I2=99.4%, Tau2=0.0227, p<0.0001				
Chai (2011)	192	781	24.6 (21.6-27.8)	2.4
Chen (2013)	308	2682	11.5 (10.3-12.8)	2.5
Cheung (2016)	162	479	33.8 (29.6-38.3)	2.4
Guo (2019)	71	155	45.8 (37.8-54.0)	2.2
He (2015)	256	754	34.0 (30.6-37.5)	2.4
Hou H (2018)	325	3005	10.8 (9.7-12.0)	2.5
Hou Y (2018)	199	1549	12.8 (11.2-14.6)	2.5
Hu (2012)	49	151	32.5 (25.1-40.5)	2.3
Hua (2008)	10	37	27.0 (13.8-44.1)	1.8
Jiang (2011)	851	2810	30.3 (28.6-32.0)	2.5
Jin (2014)	218	524	41.6 (37.3-46.0)	2.4
Li (2018)	109	359	30.4 (25.6-35.4)	2.4
Lin (2018)	57	511	11.2 (8.6-14.2)	2.4
Liu (2014)	165	441	37.4 (32.9-42.1)	2.4
Liu (2015)	115	622	18.5 (15.5-21.8)	2.4
Lu (2015)	232	353	65.7 (60.5-70.7)	2.4
Niu (2010)	55	287	19.2 (14.8-24.2)	2.4
Pan (2016)	881	5494	16.0 (15.1-17.0)	2.5
Peng (2010)	210	345	60.9 (55.5-66.1)	2.4
Shen (2016)	302	1303	23.2 (20.9-25.6)	2.4
Shao (2020)	844	1454	58.0 (55.5-60.6)	2.4
Song (2008)	289	817	35.4 (32.1-38.8)	2.4
Sun (2011)	961	5460	17.6 (16.6-18.6)	2.5
Sun (2013)	96	132	72.7 (64.3-80.1)	2.3
Sun (2017)	383	2793	13.7 (12.5-15.0)	2.5
Tan (2012)	36	294	12.2 (8.7-16.5)	2.4
Tang W (2018)	347	884	39.3 (36.0-42.6)	2.4
Wang (2013)	245	923	26.5 (23.7-29.5)	2.4
Wu (2015)	434	2774	15.6 (14.3-17.1)	2.5
Xiao (2006)	107	311	34.4 (29.1-40.0)	2.4
Xu (2002)	17	129	13.2 (7.9-20.3)	2.3
Xu (2014)	157	661	23.8 (20.6-27.2)	2.4
Yang H (2013)	547	1356	40.3 (37.7-43.0)	2.4
Yang X (2007)	20	919	2.2 (1.3-3.3)	2.5
Yu (2011)	119	525	22.7 (19.2-26.5)	2.4
Zeng (2006)	92	182	50.5 (43.1-58.0)	2.3
Zhai (2005)	61	300	20.3 (15.9-25.3)	2.4
Zhang (2005)	354	774	45.7 (42.2-49.3)	2.4
Zheng (2016)	62	174	35.6 (28.5-43.2)	2.3
Zhong (2011)	71	233	30.5 (24.6-36.8)	2.3
Zhou (2003)	40	88	45.5 (34.8-56.4)	2.1
Zhou (2009)	48	747	6.4 (4.8-8.4)	2.5
**Female**	**10097**	**44572**	**29.5 (25.6-33.4)**	**100.0**
Heterogeneity: I2=99.2%, Tau2=0.0158, p<0.0001				
**Total**	**19260**	**82619**	**29.9 (27.0-32.8)**	
Heterogeneity: I2=99.3%, Tau2=0.0182, p<0.0001				

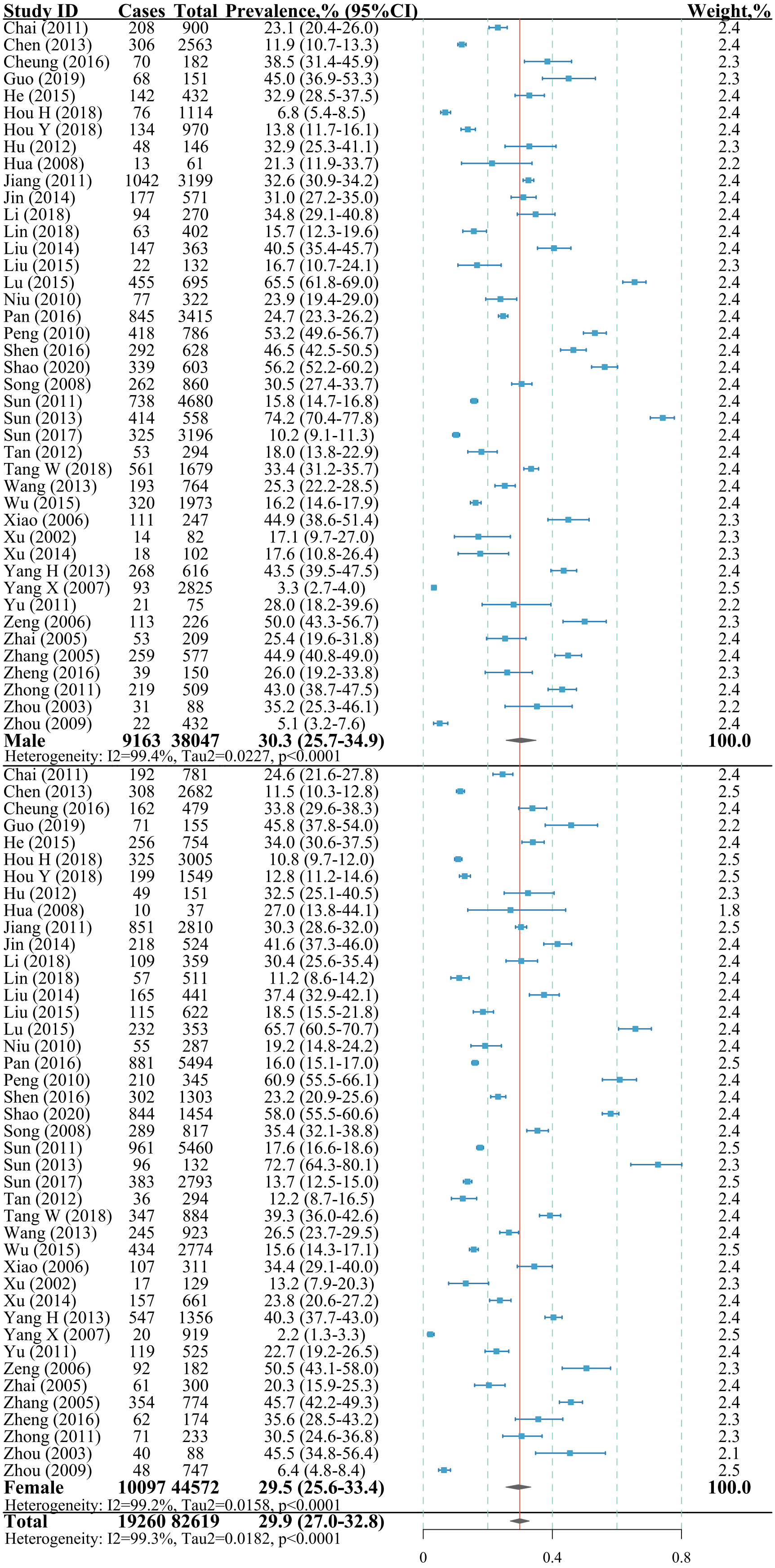

Appendix-Fig. 5 Subgroup analysis of family origin

Study ID	Cases	Total	Prevalence,% (95%CI)	Weight,%
Chai (2011)	171	732	23.4 (20.3-26.6)	4.8
Guo (2013)	201	462	43.5 (38.9-48.2)	4.7
Hou H (2018)	292	2851	10.2 (9.2-11.4)	4.8
Hu (2012)	58	170	34.1 (27.0-41.8)	4.6
Jiang (2011)	1253	3869	32.4 (30.9-33.9)	4.8
Jin (2014)	232	564	41.1 (37.0-45.3)	4.8
Li (2018)	114	391	29.2 (24.7-33.9)	4.7
Liu (2015)	71	312	22.8 (18.2-27.8)	4.7
Lu (2015)	121	169	71.6 (64.2-78.3)	4.6
Pan (2016)	1060	5030	21.1 (20.0-22.2)	4.8
Peng (2010)	345	621	55.6 (51.5-59.5)	4.8
Shao (2020)	663	1135	58.4 (55.5-61.3)	4.8
Sun (2013)	167	248	67.3 (61.1-73.1)	4.7
Sun (2017)	244	2046	11.9 (10.6-13.4)	4.8
Tang W (2018)	473	1166	40.6 (37.7-43.4)	4.8
Xu (2014)	131	562	23.3 (19.9-27.0)	4.8
Yang H (2013)	627	1568	40.0 (37.6-42.5)	4.8
Yang X (2007)	49	1783	2.7 (2.0-3.6)	4.8
Zheng (2016)	47	180	26.1 (19.9-33.2)	4.7
Zhong (2011)	157	334	47.0 (41.6-52.5)	4.7
Zhou (2009)	33	611	5.4 (3.7-7.5)	4.8
**Rural**	**6509**	**24804**	**33.6 (26.4-40.7)**	**100.0**
Heterogeneity: I2=99.6%, Tau2=0.0272, p<0.0001				
Chai (2011)	229	949	24.1 (21.4-27.0)	4.8
Guo (2013)	128	283	45.2 (39.3-51.2)	4.7
Hou H (2018)	109	1268	8.6 (7.1-10.3)	4.8
Hu (2012)	39	125	31.2 (23.2-40.1)	4.6
Jiang (2011)	610	2140	28.5 (26.6-30.5)	4.8
Jin (2014)	163	531	30.7 (26.8-34.8)	4.8
Li (2018)	89	238	37.4 (31.2-43.9)	4.7
Liu (2015)	66	443	14.9 (11.7-18.6)	4.8
Lu (2015)	566	879	64.4 (61.1-67.6)	4.8
Pan (2016)	587	3558	16.5 (15.3-17.8)	4.8
Peng (2010)	264	484	54.5 (50.0-59.0)	4.8
Shao (2020)	520	922	56.4 (53.1-59.6)	4.8
Sun (2013)	328	442	74.2 (69.9-78.2)	4.8
Sun (2017)	464	3941	11.8 (10.8-12.8)	4.8
Tang W (2018)	444	1397	31.8 (29.3-34.3)	4.8
Xu (2014)	41	190	21.6 (16.0-28.1)	4.7
Yang H (2013)	187	403	46.4 (41.5-51.4)	4.7
Yang X (2007)	64	1961	3.3 (2.5-4.1)	4.8
Zheng (2016)	54	144	37.5 (29.6-45.9)	4.6
Zhong (2011)	133	408	32.6 (28.1-37.4)	4.8
Zhou (2009)	37	567	6.5 (4.6-8.9)	4.8
**Urban**	**5122**	**21273**	**32.2 (25.0-39.4)**	**100.0**
Heterogeneity: I2=99.6%, Tau2=0.0277, p<0.0001				
**Total**	**11631**	**46077**	**32.9 (28.0-37.7)**	
Heterogeneity: I2=99.6%, Tau2=0.0251, p<0.0001				

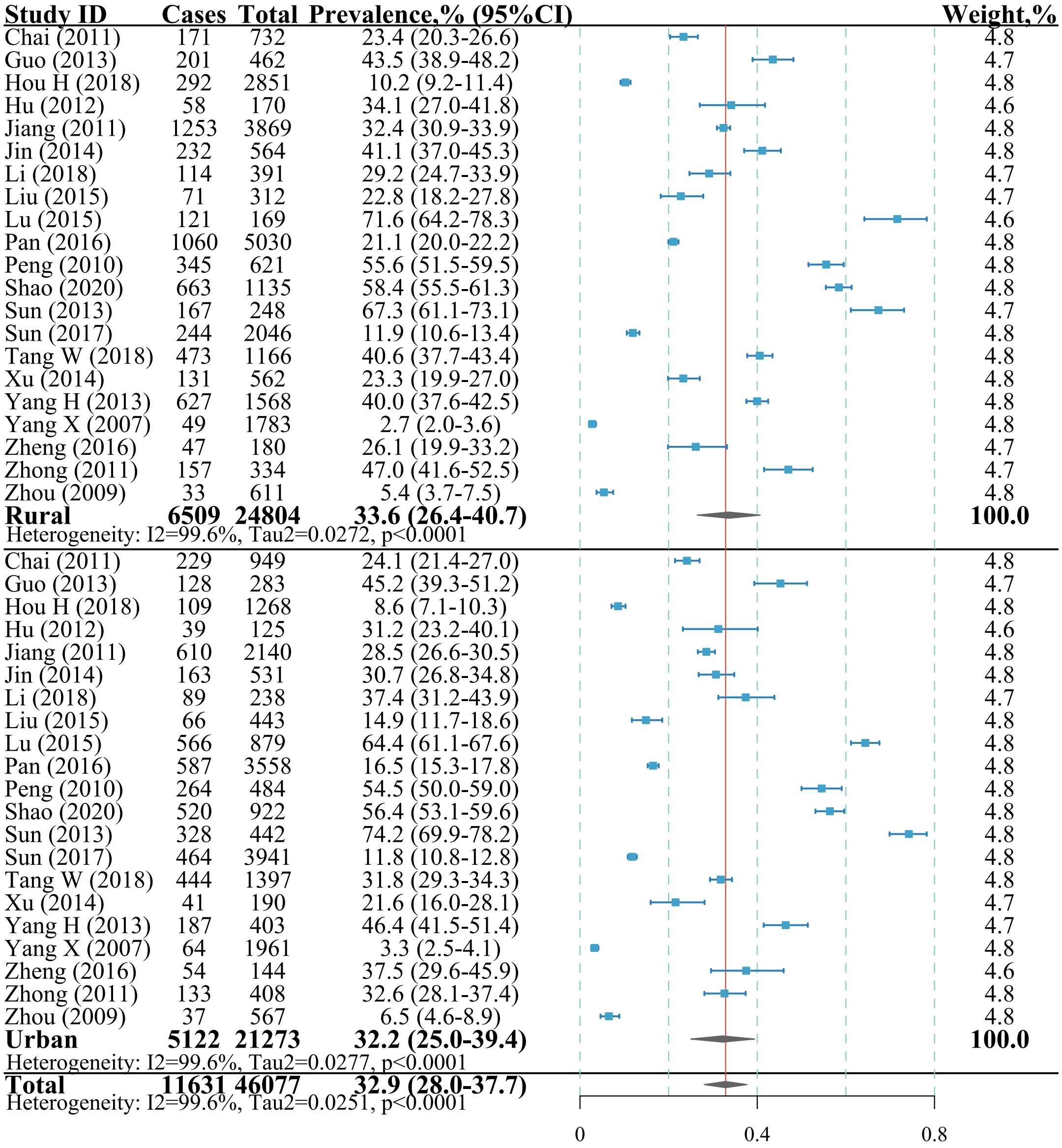

Appendix-Fig. 6 Subgroup analysis of academic grade

Study ID	Cases	Total	Prevalence,% (95%CI)	Weight,%
Chen (2013)	427	3673	11.6 (10.6-12.7)	5.6
Cheung (2016)	163	456	35.7 (31.3-40.3)	5.5
He (2015)	280	804	34.8 (31.5-38.2)	5.6
Hou H (2018)	237	1653	14.3 (12.7-16.1)	5.6
Hu (2012)	94	280	33.6 (28.1-39.4)	5.4
Jiang (2011)	1064	3247	32.8 (31.2-34.4)	5.6
Lin (2018)	93	727	12.8 (10.5-15.4)	5.6
Liu (2014)	211	529	39.9 (35.7-44.2)	5.5
Shao (2020)	881	1493	59.0 (56.5-61.5)	5.6
Sun (2011)	981	4625	21.2 (20.0-22.4)	5.6
Sun (2017)	379	3209	11.8 (10.7-13.0)	5.6
Wu (2007)	217	926	23.4 (20.7-26.3)	5.6
Xiao (2006)	138	322	42.9 (37.4-48.5)	5.4
Xu (2014)	115	484	23.8 (20.0-27.8)	5.5
Zhang (2005)	384	853	45.0 (41.6-48.4)	5.6
Zheng (2016)	71	220	32.3 (26.1-38.9)	5.3
Zhong (2011)	227	537	42.3 (38.1-46.6)	5.5
Zhou (2009)	53	813	6.5 (4.9-8.4)	5.6
**Non-freshmen**	**6015**	**24851**	**29.0 (22.9-35.1)**	**100.0**
Heterogeneity: I2=99.4%, Tau2=0.0171, p<0.0001				
Chen (2013)	190	1572	12.1 (10.5-13.8)	6.0
Cheung (2016)	69	205	33.7 (27.2-40.6)	5.5
He (2015)	121	364	33.2 (28.4-38.3)	5.7
Hou H (2018)	164	2466	6.7 (5.7-7.7)	6.0
Hu (2012)	3	12	25.0 (5.5-57.2)	2.4
Jiang (2011)	829	2762	30.0 (28.3-31.8)	6.0
Lin (2018)	27	186	14.5 (9.8-20.4)	5.7
Liu (2014)	101	275	36.7 (31.0-42.7)	5.6
Shao (2020)	302	564	53.5 (49.3-57.7)	5.8
Sun (2011)	718	5515	13.0 (12.1-13.9)	6.0
Sun (2017)	329	2777	11.8 (10.7-13.1)	6.0
Wu (2007)	86	408	21.1 (17.2-25.4)	5.8
Xiao (2006)	80	236	33.9 (27.9-40.3)	5.5
Xu (2014)	60	279	21.5 (16.8-26.8)	5.7
Zhang (2005)	229	498	46.0 (41.5-50.5)	5.8
Zheng (2016)	30	104	28.8 (20.4-38.6)	5.1
Zhong (2011)	63	205	30.7 (24.5-37.5)	5.5
Zhou (2009)	17	365	4.7 (2.7-7.4)	6.0
**Freshmen**	**3418**	**18793**	**25.1 (20.2-30.1)**	**100.0**
Heterogeneity: I2=98.8%, Tau2=0.0106, p<0.0001				
**Total**	**9433**	**43644**	**27.1 (23.2-31.0)**	
Heterogeneity: I2=99.2%, Tau2=0.0137, p<0.0001				

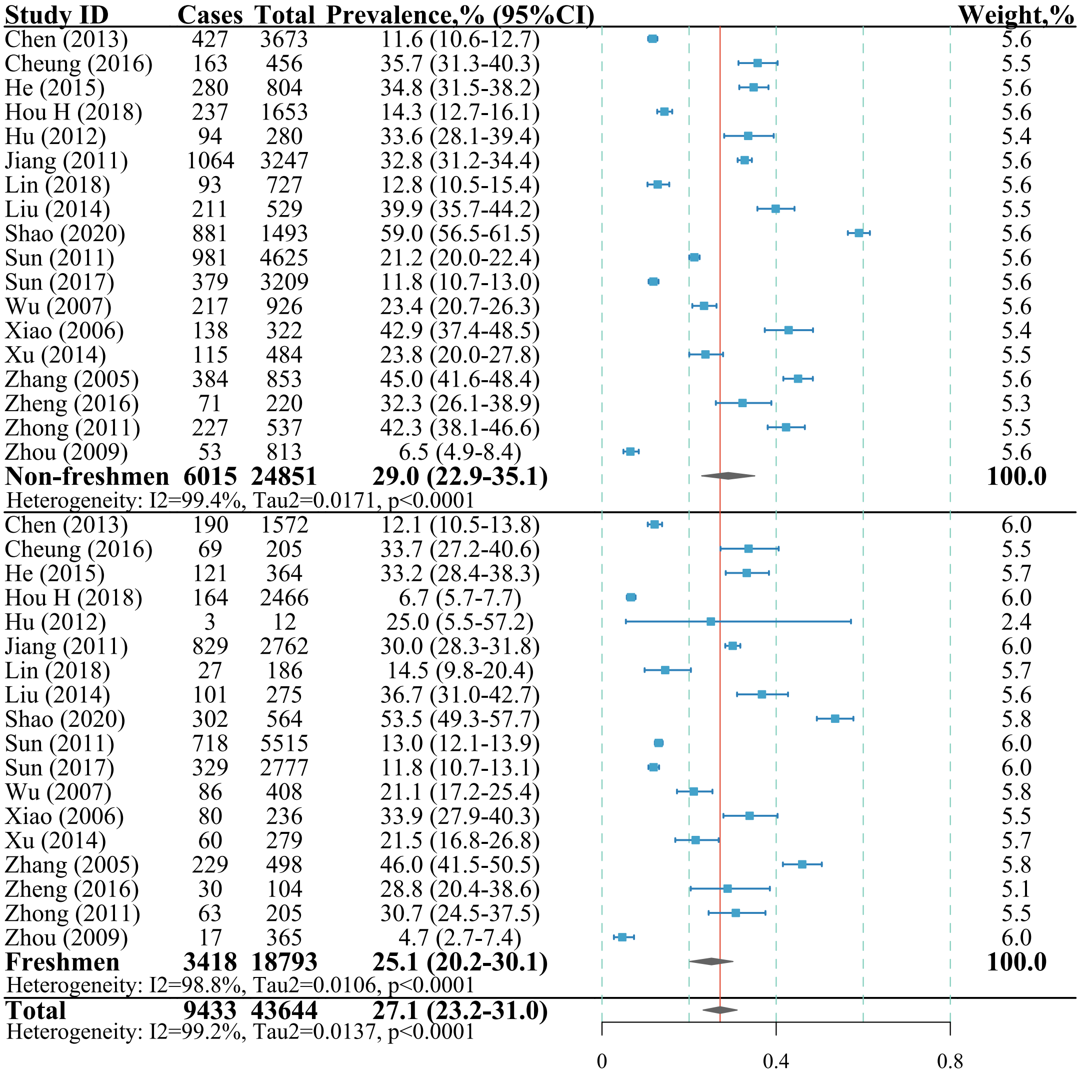

Appendix-Fig. 7 Subgroup analysis of only-child or not

Study ID	Cases	Total	Prevalence,% (95%CI)	Weight,%
He (2015)	132	363	11.8 (10.7-13.0)	12.4
Hou Y (2018)	263	1900	23.4 (20.7-26.3)	12.6
Li (2018)	125	417	42.9 (37.4-48.5)	12.4
Liu (2015)	76	352	23.8 (20.0-27.8)	12.4
Shao (2020)	777	1342	45.0 (41.6-48.4)	12.6
Shen (2016)	281	961	32.3 (26.1-38.9)	12.5
Tang W (2018)	406	1112	42.3 (38.1-46.6)	12.5
Xu (2014)	156	660	6.5 (4.9-8.4)	12.5
**Not an only-child**	**2216**	**7107**	**31.1 (19.6-42.7)**	**100.0**
Heterogeneity: I2=99.2%, Tau2=0.0276, p<0.0001				
He (2015)	267	789	11.8 (10.7-13.1)	12.6
Hou Y (2018)	70	619	21.1 (17.2-25.4)	12.7
Li (2018)	78	212	33.9 (27.9-40.3)	12.2
Liu (2015)	61	403	21.5 (16.8-26.8)	12.6
Shao (2020)	406	715	46.0 (41.5-50.5)	12.6
Shen (2016)	313	970	28.8 (20.4-38.6)	12.7
Tang W (2018)	502	1451	30.7 (24.5-37.5)	12.7
Xu (2014)	18	99	4.7 (2.7-7.4)	12.0
**Only-child**	**1715**	**5258**	**29.9 (19.5-40.3)**	**100.0**
Heterogeneity: I2=98.7%, Tau2=0.0220, p<0.0001				
**Total**	**3931**	**12365**	**30.5 (23.1-38.0)**	
Heterogeneity: I2=98.9%, Tau2=0.0226, p<0.0001				

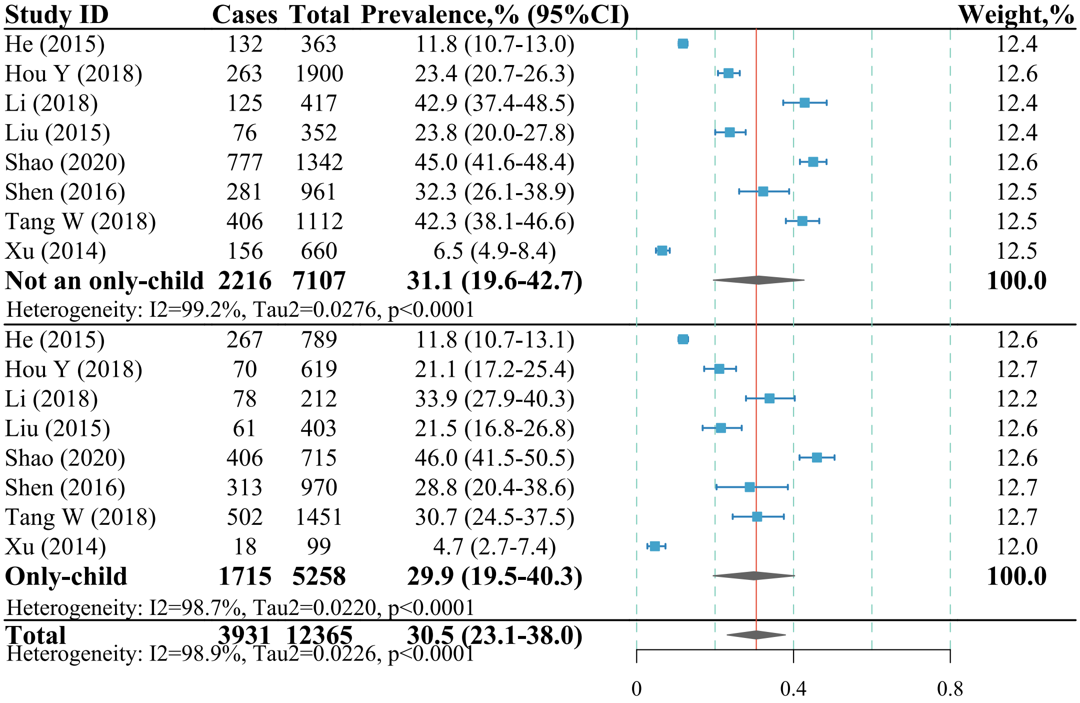

Appendix-Fig. 8 Subgroup analysis of ethnic group

Study ID	Cases	Total	Prevalence,% (95%CI)	Weight,%
Chen (2013)	50	367	13.6 (10.3-17.6)	14.9
He (2015)	53	119	44.5 (35.4-53.9)	14.1
Liu (2015)	16	82	19.5 (11.6-29.7)	14.2
Lu (2015)	41	56	73.2 (59.7-84.2)	13.5
Pan (2016)	147	771	19.1 (16.4-22.0)	15.0
Shao (2020)	76	131	58.0 (49.1-66.6)	14.2
Yang H (2013)	50	111	45.0 (35.6-54.8)	14.1
**Others**	**433**	**1637**	**38.4 (25.1-51.7)**	**100.0**
Heterogeneity: I2=97.2%, Tau2=0.0305, p<0.0001				
Chen (2013)	568	4878	11.6 (10.8-12.6)	14.3
He (2015)	347	1049	33.1 (30.2-36.0)	14.3
Liu (2015)	121	673	18.0 (15.1-21.1)	14.2
Lu (2015)	646	992	65.1 (62.1-68.1)	14.2
Pan (2016)	1546	7996	19.3 (18.5-20.2)	14.3
Shao (2020)	1107	1926	57.5 (55.2-59.7)	14.3
Yang H (2013)	765	1860	41.1 (38.9-43.4)	14.3
**Han**	**5100**	**19374**	**35.1 (22.1-48.0)**	**100.0**
Heterogeneity: I2=99.8%, Tau2=0.0304, p<0.0001				
**Total**	**5533**	**21011**	**36.7 (27.7-45.7)**	
Heterogeneity: I2=99.6%, Tau2=0.0286, p<0.0001				

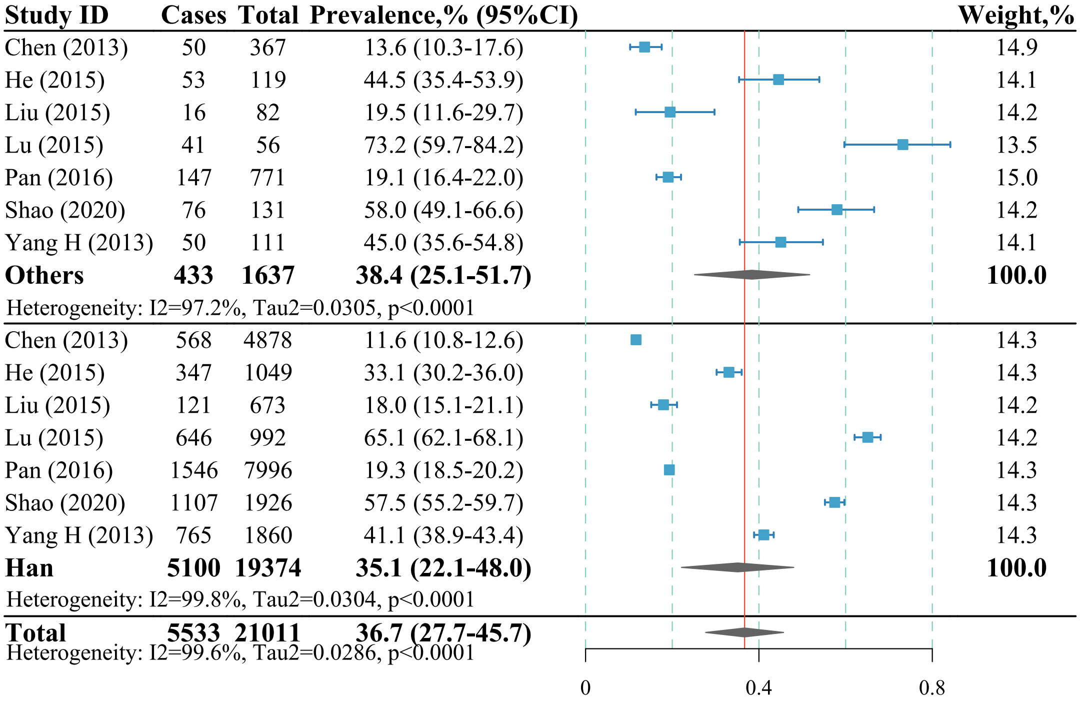

Appendix-Fig. 9 Subgroup analysis of medical students or not

Study ID	Cases	Total	Prevalence,% (95%CI)	Weight,%
Chen (2013)	50	579	13.6 (10.3-17.6)	17.4
Hou H (2018)	285	2460	44.5 (35.4-53.9)	17.6
Pan (2016)	809	4111	19.5 (11.6-29.7)	17.6
Shen (2016)	461	1434	73.2 (59.7-84.2)	17.4
Zeng (2006)	105	197	19.1 (16.4-22.0)	15.5
Zheng (2016)	36	107	58.0 (49.1-66.6)	14.4
**Medical**	**1746**	**8888**	**25.7 (17.9-33.6)**	**100.0**
Heterogeneity: I2=98.8%, Tau2=0.0091, p<0.0001				
Chen (2013)	567	4666	11.6 (10.8-12.6)	17.9
Hou H (2018)	116	1659	33.1 (30.2-36.0)	17.8
Pan (2016)	893	4801	18.0 (15.1-21.1)	17.9
Shen (2016)	133	497	65.1 (62.1-68.1)	16.7
Zeng (2006)	100	211	19.3 (18.5-20.2)	14.6
Zheng (2016)	65	217	57.5 (55.2-59.7)	15.1
**Non-medical**	**1874**	**12051**	**22.7 (16.7-28.6)**	**100.0**
Heterogeneity: I2=98.6%, Tau2=0.0051, p<0.0001				
**Total**	**3620**	**20939**	**24.0 (19.6-28.5)**	
Heterogeneity: I2=98.7%, Tau2=0.0058, p<0.0001				

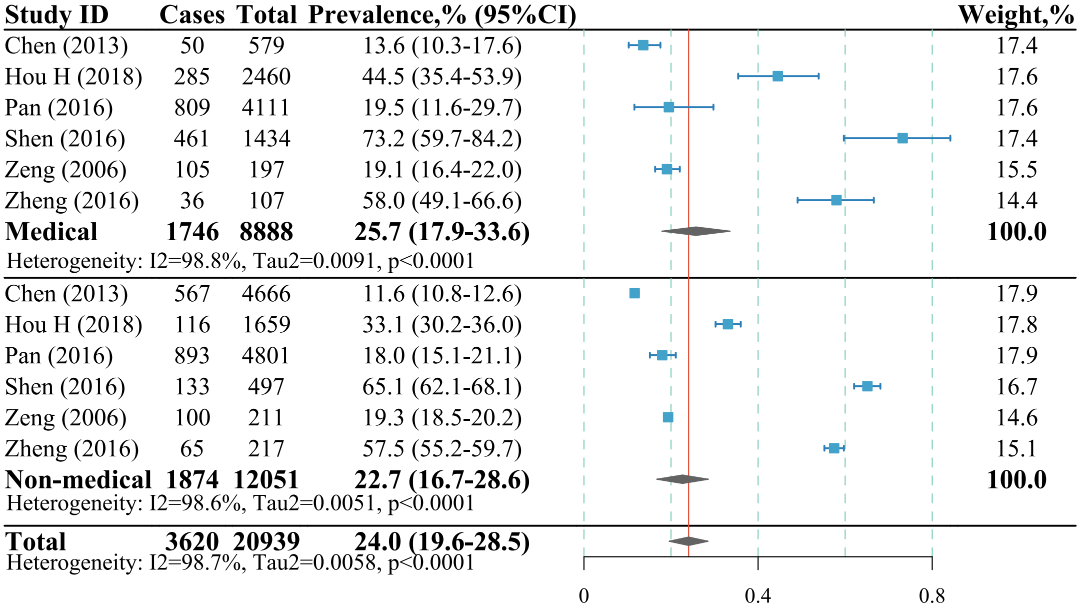

Appendix-Fig. 10 Subgroup analysis of dating relationship

Study ID	Cases	Total	Prevalence,% (95%CI)	Weight,%
He (2015)	256	776	33.0 (29.7-36.4)	20.7
Liu (2014)	229	546	41.9 (37.8-46.2)	20.0
Shen (2016)	419	1422	29.5 (27.1-31.9)	21.3
Zheng (2016)	71	222	32.0 (25.9-38.6)	18.1
Zhong (2011)	232	512	45.3 (40.9-49.7)	19.9
**Without a bf/gf friend**	**1207**	**3478**	**36.3 (30.1-42.5)**	**100.0**
Heterogeneity: I2=92.8%, Tau2=0.0046, p<0.0001				
He (2015)	142	384	37.0 (32.1-42.0)	22.4
Liu (2014)	83	258	32.2 (26.5-38.2)	20.0
Shen (2016)	175	509	34.4 (30.3-38.7)	24.4
Zheng (2016)	30	102	29.4 (20.8-39.3)	13.0
Zhong (2011)	58	230	25.2 (19.7-31.3)	20.2
**Has a bf/gf friend**	**488**	**1483**	**32.0 (27.9-36.1)**	**100.0**
Heterogeneity: I2=63.8%, Tau2=0.0014, p=0.0261				
**Total**	**1695**	**4961**	**34.2 (30.4-38.0)**	
Heterogeneity: I2=86.7%, Tau2=0.0031, p<0.0001				

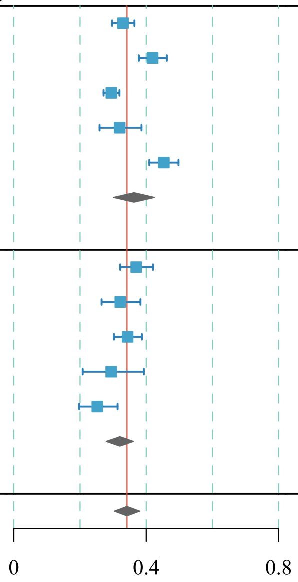

Appendix-Fig. 11 Subgroup analysis of religious belief

Study ID	Cases	Total	Prevalence,% (95%CI)	Weight,%
Cheung (2016)	62	182	33.0 (29.7-36.4)	25.9
He (2015)	48	98	41.9 (37.8-46.2)	25.1
Liu (2015)	15	50	29.5 (27.1-31.9)	24.2
Lu (2015)	39	49	32.0 (25.9-38.6)	24.7
**Religious**	**164**	**379**	**48.1 (27.6-68.6)**	**100.0**
Heterogeneity: I2=94.2%, Tau2=0.0409, p<0.0001				
Cheung (2016)	170	479	37.0 (32.1-42.0)	24.9
He (2015)	354	1059	32.2 (26.5-38.2)	25.0
Liu (2015)	122	705	34.4 (30.3-38.7)	25.0
Lu (2015)	648	999	29.4 (20.8-39.3)	25.0
**Irreligious**	**1294**	**3242**	**37.8 (16.8-58.7)**	**100.0**
Heterogeneity: I2=99.4%, Tau2=0.0453, p<0.0001				
**Total**	**1458**	**3621**	**42.8 (28.2-57.3)**	
Heterogeneity: I2=98.8%, Tau2=0.0425, p<0.0001				

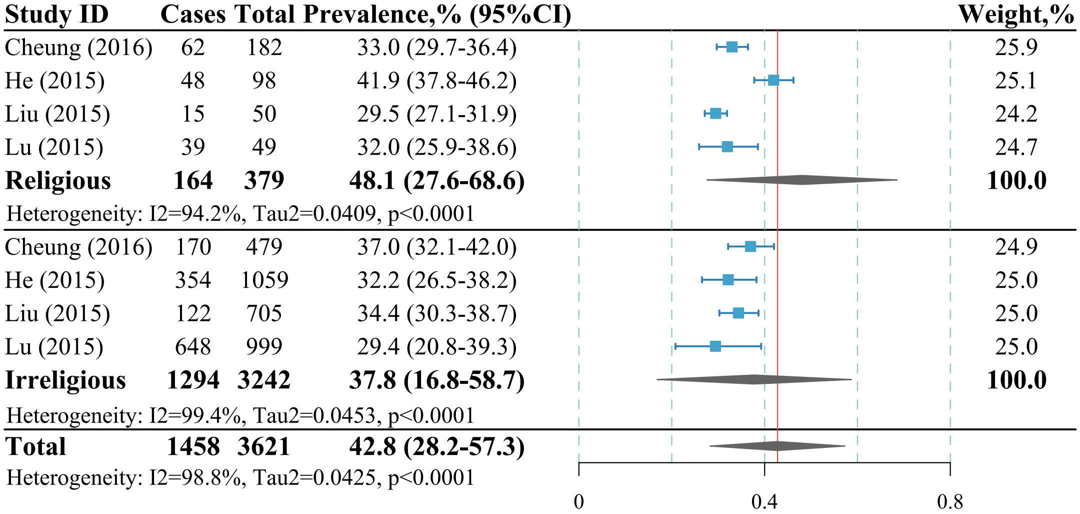

Appendix-Fig. 12 Subgroup analysis of left-behind experiences on childhood

Study ID	Cases	Total	Prevalence,% (95%CI)	Weight,%
Han (2018)	167	367	45.5 (40.3-50.8)	24.8
Hou Y (2018)	114	798	14.3 (11.9-16.9)	25.4
Jiang (2011)	177	529	33.5 (29.4-37.7)	25.1
Liu (2020)	93	311	29.9 (24.9-35.3)	24.8
**Experienced**	**551**	**2005**	**30.7 (16.4-44.9)**	**100.0**
Heterogeneity: I2=98.1%, Tau2=0.0206, p<0.0001				
Han (2018)	98	421	23.3 (19.3-27.6)	24.3
Hou Y (2018)	219	1721	12.7 (11.2-14.4)	25.3
Jiang (2011)	1716	5840	29.4 (28.2-30.6)	25.3
Liu (2020)	207	1194	17.3 (15.2-19.6)	25.1
**No experiences**	**2240**	**9176**	**20.7 (11.4-30.0)**	**100.0**
Heterogeneity: I2=99.0%, Tau2=0.0089, p<0.0001				
**Total**	**2791**	**11181**	**25.5 (18.7-32.4)**	
Heterogeneity: I2=98.5%, Tau2=0.0094, p<0.0001				

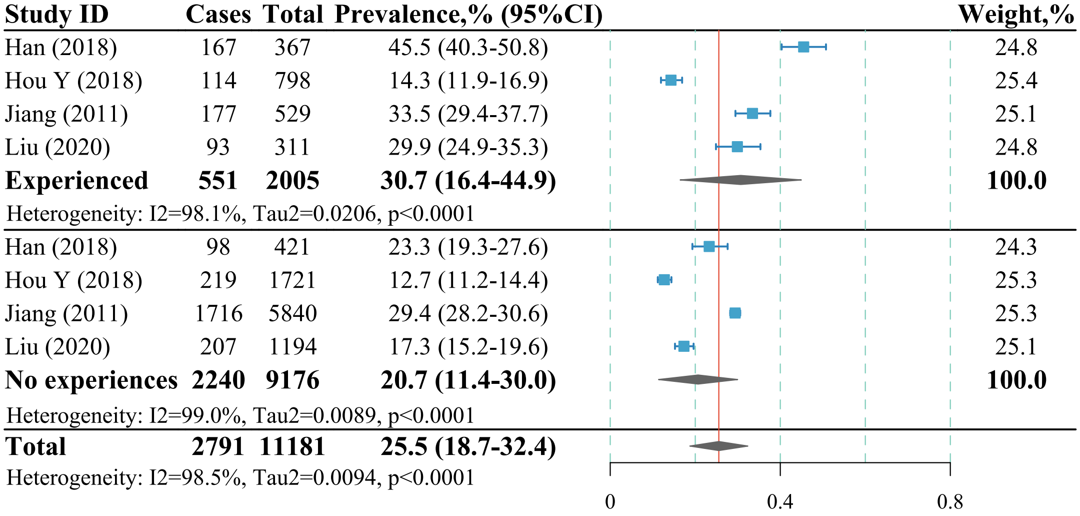

Appendix-Fig. 13 Subgroup analysis of educational level of father

Study ID	Cases	Total	Prevalence,% (95%CI)	Weight,%
Hou Y (2018)	179	1197	45.5 (40.3-50.8)	25.3
Jiang (2011)	862	2698	14.3 (11.9-16.9)	25.4
Pan (2016)	983	4361	33.5 (29.4-37.7)	25.6
Xu (2014)	114	439	29.9 (24.9-35.3)	23.7
**≤9 years**	**2138**	**8695**	**23.8 (16.8-30.9)**	**100.0**
Heterogeneity: I2=98.1%, Tau2=0.0050, p<0.0001				
Hou Y (2018)	154	1322	23.3 (19.3-27.6)	25.6
Jiang (2011)	484	1783	12.7 (11.2-14.4)	25.4
Pan (2016)	758	4621	29.4 (28.2-30.6)	25.9
Xu (2014)	60	312	17.3 (15.2-19.6)	23.1
**>9 years**	**1456**	**8038**	**18.6 (12.4-24.8)**	**100.0**
Heterogeneity: I2=97.7%, Tau2=0.0038, p<0.0001				
**Total**	**3594**	**16733**	**21.2 (16.4-26.0)**	
Heterogeneity: I2=98.3%, Tau2=0.0047, p<0.0001				

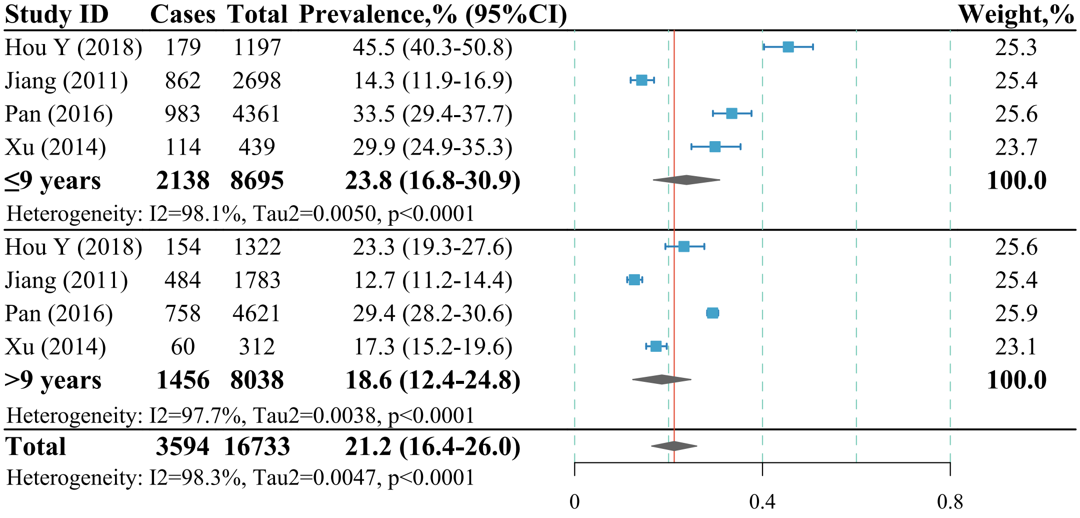

Appendix-Fig. 14 Subgroup analysis of educational level of mother

Study ID	Cases	Total	Prevalence,% (95%CI)	Weight,%
Hou Y (2018)	217	1634	13.3 (11.7-15.0)	33.5
Jiang (2011)	1070	3435	31.1 (29.6-32.7)	33.6
Xu (2014)	141	574	24.6 (21.1-28.3)	32.9
**≤9 years**	**1428**	**5643**	**23.0 (10.3-35.7)**	**100.0**
Heterogeneity: I2=99.2%, Tau2=0.0124, p<0.0001				
Hou Y (2018)	115	868	13.2 (11.1-15.7)	34.5
Jiang (2011)	283	1079	26.2 (23.6-29.0)	34.3
Xu (2014)	34	180	18.9 (13.5-25.4)	31.2
**>9 years**	**432**	**2127**	**19.5 (10.1-28.8)**	**100.0**
Heterogeneity: I2=96.3%, Tau2=0.0065, p<0.0001				
**Total**	**1860**	**7770**	**21.2 (13.7-28.8)**	
Heterogeneity: I2=98.4%, Tau2=0.0086, p<0.0001				

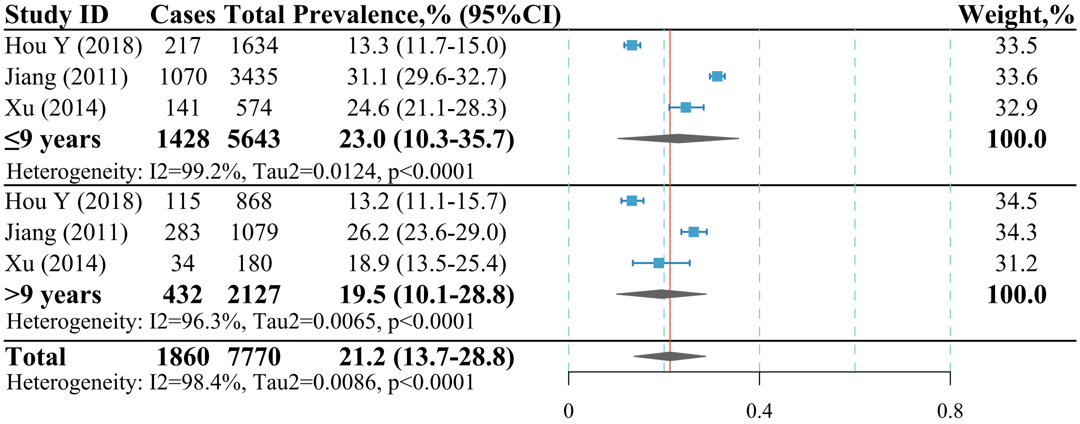

Appendix-Fig. 15 Subgroup analysis of smoking or not

Study ID	Cases	Total	Prevalence,% (95%CI)	Weight,%
Cai (2017)	85	207	41.1 (34.3-48.1)	50.3
Cheung (2016)	1	5	20.0 (0.5-71.6)	12.8
Zheng (2016)	9	37	24.3 (11.8-41.2)	36.9
**Smoker**	**95**	**249**	**32.2 (17.9-46.5)**	**100.0**
Heterogeneity: I2=63.2%, Tau2=0.0012, p=0.0186				
Cai (2017)	336	1120	30.0 (27.3-32.8)	41.8
Cheung (2016)	231	656	35.2 (31.6-39.0)	34.5
Zheng (2016)	92	287	32.1 (26.7-37.8)	23.7
**Non-smoker**	**659**	**2063**	**32.3 (28.8-35.8)**	**100.0**
Heterogeneity: I2=60.7%, Tau2=0.0006, p=0.0787				
**Total**	**754**	**2312**	**33.2 (29.2-37.1)**	
Heterogeneity: I2=63.8%, Tau2=0.0094, p=0.0630				

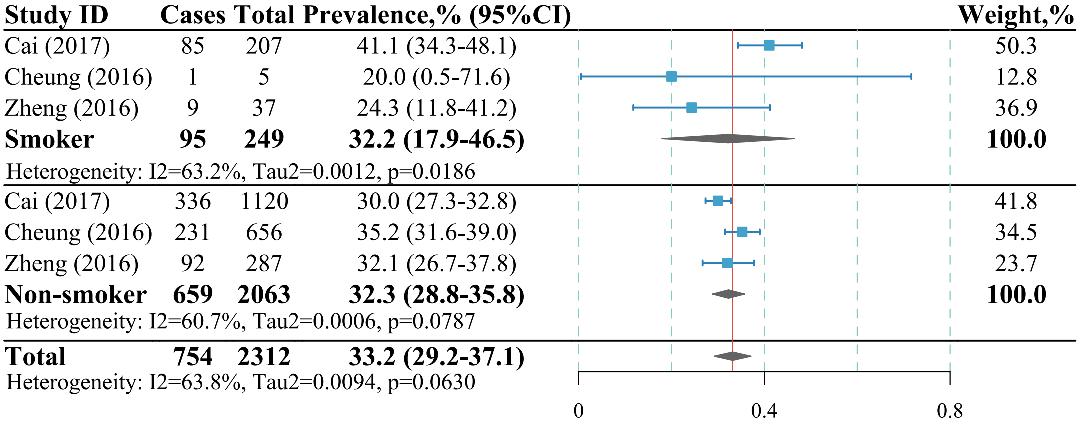

References

1 Cai, L. *et al.* Social smoking and mental health among Chinese male college students. *Am J Health Promot* **31**, 226-231, doi:10.4278/ajhp.141001-QUAN-494 (2017).

2 Chai, X. Investigation and research on depression of college students in our university. *Journal of Yangtze University (Nat Sci Edit)* **8**, 198-200, doi:10.3969/j.issn.1673-1409.2011.01.074 (2011).

3 Chan, D. W. Coping with depressed mood among Chinese medical students in Hong-kong. *Journal of Affective Disorders* **24**, 109-116, doi:10.1016/0165-0327(92)90025-2 (1992).

4 Chang, S. M., Law, D. W. & Chang, H. K. The impact of personality on depression among university students in Taiwan. *Chang Gung Med J* **34**, 528-535 (2011).

5 Chen, L. *et al.* Depression among Chinese university students: prevalence and socio-demographic correlates. *PLOS ONE* **8**, doi:10.1371/journal.pone.0058379 (2013).

6 Chen, C. J. *et al.* The prevalence and related factors of depressive symptoms among junior college nursing students: a cross-sectional study. *Journal of Psychiatric and Mental Health Nursing* **22**, 590-598, doi:10.1111/jpm.12252 (2015).

7 Chen, L. *et al.* Relationship between personality characteristics, constitutions of traditional chinese medicine and depression in undergraduate *Chinese General Practice* **19**, 4124-4129, doi:10.3969/j.issn.1007-9572.2016.33.021 (2016).

8 Cheung, T. *et al.* Depression, anxiety and symptoms of stress among baccalaureate nursing students in Hong Kong: A cross-sectional study. *Int J Environ Res Public Health* **13**, doi:10.3390/ijerph13080779 (2016).

9 Chou, W.-P., Yen, C.-F. & Liu, T.-L. Predicting effects of psychological inflexibility/experiential avoidance and stress coping strategies for internet addiction, significant depression, and suicidality in college students: A prospective study. *International journal of environmental research and public health* **15**, 788, doi:10.3390/ijerph15040788 (2018).

10 Deng, F. *et al.* Early menarche and psychopathological symptoms in young Chinese women. *Journal of Womens Health* **20**, 207-213, doi:10.1089/jwh.2010.2102 (2011).

11 Dong, Y. & Li, H. The relationship between social support and depressive symptoms among the college students of Liaoning, China: a moderated mediated analysis. *Psychology Health & Medicine*, doi:10.1080/13548506.2019.1679844 %/ routledge journals, taylor & francis ltd (2019).

12 Du, Z. & Wang, K. An epidemiological study on depression in 1597 undergraduates. *Chinese Journal of Behavioral Medical Science* **8**, 14-15 (1999).

13 Fang, H., Cui, W. & Fan, H. The relationship between frustration events, anti-frustration ability and college studentg depressions *China Journal of Health*

Psychology **25**, 1089-1092 (2017).
14 Feng, G., Chen, J. & Yang, X. Study on the status and quality of sleep-related influencing factors in medical college students. *Chinese Journal of Epidemiology* **26**, 328-331 (2005).
15 Feng, Q., Zhang, Q.-l., Du, Y., Ye, Y.-l. & He, Q.-q. Associations of physical activity, screen time with depression, anxiety and sleep quality among Chinese college freshmen. *PLOS ONE* **9**, doi:10.1371/journal.pone.0100914 (2014).
16 Fu, G. The relationship between undergraduate students' social supports and depression. *Medicine and Society* **23**, 83-84 (2010).
17 Gao, G. *et al.* Relationship between depression and network *Pratical Preventive Medicine* **15**, 301-303, doi:10.3969/j.issn.1006-3110.2008.02.003 (2008).
18 Gao, T. *et al.* Prevalence and correlates of suicidal behaviors among college students in northeastern China: a cross-sectional study. *Psychiatr Q* **89**, 359-370, doi:10.1007/s11126-017-9540-9 (2018).
19 Guo, Y., Zhang, N. & Zhang, J. Comparation of positive psychological quality and its influencing factors among nursing students from rural and urban areas. *Chinese Journal of Public Health* **29**, 1041-1045, doi:10.11847/zgggws2013-29-07-33 (2013).
20 Guo, M. *et al.* Correlation analysis of acne incidence, emotion and physical constitution among college students in Fuzhou. *Guiding Journal of Traditional Chinese Medicine and Pharmacy* **25**, 85-88 (2019).
21 Guo, F., Tian, Y., Cui, Y. & Huang, C. Night-eating syndrome and depressive symptoms in college freshmen: Fitness improvement tactics in youths (FITYou) project. *Psychology Research and Behavior Management* **13**, 185-191, doi:10.2147/PRBM.S234025 %/ © 2020 Guo et al. (2020).
22 Hall, B. J. *et al.* An evaluation of a low intensity mHealth enhanced mindfulness intervention for Chinese university students: A randomized controlled trial. *Psychiatry Research* **270**, 394-403, doi:10.1016/j.psychres.2018.09.060 (2018).
23 Han, H. Analysis and countermeasures of SDS for students majoring in arts in universities. *Dushi Jiajiao*, 65 (2011).
24 Han, Y. *et al.* Reason analysis and prevention of depression among students in a medical college. *Journal of Shenyang Medical College* **17**, 56-57, doi:10.3969/j.issn.1008-2344.2015.01.021 (2015).
25 Han, L., Zhao, S. Y., Pan, X. Y. & Liao, C. J. The impact of students with left-behind experiences on childhood: The relationship between negative life events and depression among college students in China. *Int J Soc Psychiatry* **64**, 56-62, doi:10.1177/0020764017739332 (2018).
26 He, H. & Luo, Y. Analysis on depression status and influence factors of university students in Beijing. *Modern Preventive Medicine* **42**, 1261-1264 (2015).
27 Hou, X., Chen, K. & Yu, Q. Investigation and analysis of mental health status of freshmen in a medical college. *Guide of China Medicine* **12**, 358-359 (2014).
28 Hou, H. *et al.* Suboptimal health status and psychological symptoms among Chinese college students: a perspective of predictive, preventive and

personalised health. *EPMA Journal* **9**, 367-377, doi:10.1007/s13167-018-0148-4 (2018).
29 Hou, Y., Chen, Y. & Zhang, X. Revalence of depressive symptom and associated family factors among college students. *Modern Preventive Medicine* **45**, 471-475 (2018).
30 Hu, L. & Tang, X. Study on the relationship between depression and family closeness and adaptability in college students. *Journal of Shangrao Normal University* **32**, 91-94, doi:10.3969/j.issn.1004-2237.2012.01.020 (2012).
31 Hua, Y. Analysis of depression and mental health of college students. *Scientific Information (Academic Research)*, 356-358 (2008).
32 Jiang, D., Yao, R., Yuan, Z. & Tao, F. Analysis of depression and anxiety among bengbu university students and its influencing factors. *Journal of Hygiene Research* **40**, 541-543 (2011).
33 Jin, P., Yu, J. & Lu, S. Analysis of depression of students of three private colleges in Nanjing city. *Occupation and Health* **30**, 3586-3588 (2014).
34 Kang, C. A study on the effect of psychological nursing intervention on college students' depression. *Guide of China Medicine* **15**, 290-291 (2017).
35 Lei, X. *et al.* Measurement equivalence of the 10-item ruminative response scale across groups with screen-negative or screen-positive depressive symptoms. *Chinese Journal of Clinical Psychology* **26**, 39-42, doi:10.16128/j.cnki.1005-3611.2018.01.009 (2018).
36 Li, L., Wan, L. & Zhao, S. Characteristics and influencing factors of depression of college students. *Journal of Qiannan Normal University for Nationalities* **38**, 81-84 (2018).
37 Lin, F. *et al.* Depression and its influencing factors in medical students. *China Journal of Health Psychology* **26**, 622-627 (2018).
38 Liu, X., Tang, M., Hu, L., Wang, A. & Li, C. A study on the correlation between anxiety, depression and sleep quality in college students. *Chinese Mental Health Journal* **11**, 26-28 (1997).
39 Liu, B. *et al.* Study about related factors of depression and anxiety in university freshmen in Shandong province. *Journal of Psychiatry* **24**, 100-102 (2011).
40 Liu, A. Analysis of depression symptoms and its influencing factors in college students of Hunan university of science and technology. *Journal of Hygiene Research* **43**, 508-511 (2014).
41 Liu, Y. *et al.* A survey on depression in college students and influencing factors *Chinese Journal of General Practice* **13**, 91-93 (2015).
42 Liu, Y. *et al.* Problematic smart phone use and its relation with anxiety and depression: a survey in a large sample of undergraduates *Chinese Journal of Psychiatry* **50**, 270-275, doi:10.3760/cma.j.issn.1006-7884.2017.04.008 (2017).
43 Liu, X., Ping, S. & Gao, W. Changes in undergraduate students' psychological well-being as they experience university life. *International Journal of Environmental Research and Public Health* **16**, doi:10.3390/ijerph16162864 (2019).
44 Liu, H. *et al.* The influence of left-behind experience on college students' mental

health: A cross-sectional comparative study. *International Journal of Environmental Research and Public Health* **17**, 1511, doi:10.3390/ijerph17051511 (2020).

45 Lu, W. *et al.* Prevalence and related risk factors of anxiety and depression among Chinese college freshmen. *Journal of Huazhong University of Science and Technology-Medical Sciences* **35**, 815-822, doi:10.1007/s11596-015-1512-4 (2015).

46 Luo, H. & Ma, J. Relation among college student's depression symptom, problem solving appraisal and perceived stress. *Chinese Journal of Clinical Psychology* **12**, 367-368 (2004).

47 Ma, H. *et al.* Study on the relationships of depressive symptoms with personality traits and coping style of undergraduates. *Chinese Journal of Health Education* **35**, 179-181, doi:10.16168/j.cnki.issn.1002-9982.2019.02.018 (2019).

48 Niu, R., Jiang, G.-l. & Fang, Z.-y. Status of coping style, anxiety and depression of college freshmen and their relationship. *Chinese Journal of Health Education* **26**, 548-550 (2010).

49 Pan, X.-F. *et al.* Prevalence of depressive symptoms and its correlates among medical students in China: a national survey in 33 universities. *Psychology Health & Medicine* **21**, 882-889, doi:10.1080/13548506.2015.1127395 (2016).

50 Peng, M. *et al.* Employment-related anxiety and depression in senior college students in China. *Zhong Nan Da Xue Xue Bao Yi Xue Ban* **35**, 194-202, doi:10.3969/j.issn.1672-7347.2010.03.002 (2010).

51 Shao, R. *et al.* Prevalence of depression and anxiety and correlations between depression, anxiety, family functioning, social support and coping styles among Chinese medical students. *BMC psychology* **8**, 1-19, doi:10.1186/s40359-020-00402-8 (2020).

52 Shen, J. *et al.* The depression condition and influencing factors of college students in Xuzhou city. *Chinese Journal of Prevention and Control of Chronic Diseases* **24**, 890-893, doi:10.16386/j.cjpccd.issn.1004-6194.2016.12.003 (2016).

53 Shi, M., Liu, L., Wang, Z. Y. & Wang, L. Prevalence of depressive symptoms and its correlations with positive psychological variables among Chinese medical students: an exploratory cross-sectional study. *BMC Psychiatry* **16**, doi:10.1186/s12888-016-0710-3 (2016).

54 Sobowale, K., Zhou, N., Fan, J., Liu, N. & Sherer, R. Depression and suicidal ideation in medical students in China: a call for wellness curricula. *Int J Med Educ* **5**, 31-36, doi:10.5116/ijme.52e3.a465 (2014).

55 Song, Y. *et al.* Depression in college: depressive symptoms and personality factors in Beijing and Hong Kong college freshmen. *Comprehensive Psychiatry* **49**, 496-502, doi:10.1016/j.comppsych.2008.02.005 (2008).

56 Sun, D., Zhu, L. & Yang, H. Survey and analyses on mental health of freshmen and seniors in medical schools. *Journal of the Fourth Military Medical University* **29**, 705-707 (2008).

57 Sun, L. *et al.* Correlations between psychological symptoms and social

relationships among medical undergraduates in Anhui province of China. *International Journal of Psychiatry in Medicine* **42**, 29-47, doi:10.2190/PM.42.1.c (2011).

58 Sun, L. Analysis of the distribution of depression among college students. *Journal of Hubei University of Economics (Humanities and Social Sciences)* **10**, 153-154 (2013).

59 Sun, X.-j., Niu, G.-f., You, Z.-q., Zhou, Z.-k. & Tang, Y. Gender, negative life events and coping on different stages of depression severity: A cross-sectional study among Chinese university students. *Journal of Affective Disorders* **209**, 177-181, doi:10.1016/j.jad.2016.11.025 (2017).

60 Tan, X., Lu, X. & Fu, Y. Investigation on anxiety and depression of vocational college students. *Daguan Weekly* **562**, 132 (2012).

61 Tang, F., Byrne, M. & Qin, P. Psychological distress and risk for suicidal behavior among university students in contemporary China. *Journal of Affective Disorders* **228**, 101-108, doi:10.1016/j.jad.2017.12.005 (2018).

62 Tang, W. & Dai, Q. Depressive symptoms among first-year Chinese undergraduates: The roles of socio-demographics, coping style, and social support. *Psychiatry Research* **270**, 89-96, doi:10.1016/j.psychres.2018.09.027 (2018).

63 Tong, L. *et al.* Mediating effect of anxiety and depression on the relationship between Attention-deficit/hyperactivity disorder symptoms and smoking/drinking. *Scientific Reports* **6**, doi:10.1038/srep21609 (2016).

64 Wang, T., Wang, N., Hu, H., Feng, Z. & Liu, Y. Relationship of depression, automatic thoughts and personality in medical college students. *Journal of Third Military Medical University* **29**, 442-444 (2007).

65 Wang, Y., Wang, H. & Li, J. Mental status of undergraduates in Shenyang *Journal of Clinical Psychiatry* **21**, 393-394 (2011).

66 Wang, Q., Wang, H. & Zhao, J. Effects of self-conceppt and coping-style on depression of undergraduates. *China Journal of Health Psychology* **20**, 273-276 (2012).

67 Wang, J. *et al.* Correlation analysis between depressed mood and life events among medical college students. *Journal of Wannan Medical College* **32**, 151-153 (2013).

68 Wang, L. *et al.* Professional identity and mental health of rural-oriented tuition-waived medical students in Anhui Province, China. *BMC Medical Education* **19**, doi:10.1186/s12909-019-1603-1 (2019).

69 Wang, M. *et al.* Prevalence and correlates of suicidal ideation among college students: A mental health survey in Jilin Province, China. *Journal of Affective Disorders* **246**, 166-173, doi:10.1016/j.jad.2018.12.055 (2019).

70 Wang, Z., Xu, S. & Duan, H. Investigation and analysis of anxiety and depression among female nursing students -- a case study of two colleges in northwest China. *Technology & Management*, 33-35 (2019).

71 Wei, Y., Yan, Y., Lin, R. & Liu, L. Correlation between depression and sleep quality among college students in Fuzhou city. *Chinese Journal of School*

Doctor **25**, 172-173 (2011).
72 Wu, Z., Zhang, H., Xu, J., Wang, J. & Wang, J. Correlation between anxiety, depression and personality among female collge students in Anhui province. *Chinese Journal of School Health* **28**, 232-234, doi:10.3969/j.issn.1000-9817.2007.03.031 (2007).
73 Wu, X., Tao, S., Zhang, Y., Zhang, S. & Tao, F. Low physical activity and high screen time can increase the risks of mental health problems and poor sleep quality among Chinese college students. *PLOS ONE* **10**, doi:10.1371/journal.pone.0119607 (2015).
74 Wu, X. *et al.* Impact of screen time on mental health problems progression in youth: a 1-year follow-up study. *BMJ Open* **6**, e011533, doi:10.1136/bmjopen-2016-011533 (2016).
75 Xi, M. Depression status of the students in Xingtai university and its cause analysis. *Journal of Xingtai University* **25**, 12-13 (2010).
76 Xiao, W., Zhou, H., Xia, Y., Yuan, J. & Yan, Q. Depression status and its risk factors among the college students. *Chinese Journal of Behavioral Medicine and Brain Science* **15**, 647-649 (2006).
77 Xiao, J. *et al.* 'Weakest Link' as a Cognitive Vulnerability Within the Hopelessness Theory of Depression in Chinese University Students. *Stress and Health* **32**, 20-27, doi:10.1002/smi.2571 (2016).
78 Xu, L., Guan, W., Wei, X., Li, W. & Sun, X. Investigation and analysis of anxiety and depression among junior medical college students. *China Journal of Health Psychology* **10**, 24-25 (2002).
79 Xu, Y., Zhu, X., Zhao, J. & Liu, L. Investigation and analysis of mental health status of 1750 college students. *Chinese Journal of Primary Medicine and Pharmacy* **10**, 113 (2003).
80 Xu, Y. *et al.* Prevalence and correlates of depression among college nursing students in China. *Nurse Educ Today* **34**, e7-12, doi:10.1016/j.nedt.2013.10.017 (2014).
81 Xu, Y., Qi, J., Yang, Y. & Wen, X. The contribution of lifestyle factors to depressive symptoms: A cross-sectional study in Chinese college students. *Psychiatry Research* **245**, 243-249, doi:10.1016/j.psychres.2016.03.009 (2016).
82 Xu, H. *et al.* Beverages consumption, sleep quality and depressive symptoms in Chinese university students: a latent variable mediation model. *Chinese Journal of School Health* **41**, 16-20, doi:10.16835/j.cnki.1000-9817.2020.01.005 (2020).
83 Yang, S., Liang, X., Li, J. & Gong, X. Investigation on depression of "left-behind" college students in a university. *Chinese Journal of School Health* **29**, 51-52 (2008).
84 Yang, C. *et al.* Analysis on suicide ideation and its risk factors among medical college students. *Chinese Journal of Social Medicine* **30**, 193-195 (2013).
85 Yang, H., Chen, Y. & Chou, Y. Epidemiologic survey on the depressive symptoms of college freshmen *Modern Preventive Medicine* **40**, 3340-3342 (2013).
86 Yang, M., Du, J. & Wang, X. Comparative research of depressive emotion

between college students and social youths with the same age as college students. *China Journal of Health Psychology* **15**, 400-402, doi:10.3969/j.issn.1005-1252.2007.05.005 (2007).

87 Yang, X. An epidemiological investigation of depression among 3744 freshmen. *China Journal of Health Psychology* **15**, 499-501 (2007).

88 Ye, Y.-l. *et al.* Associations between multiple health risk behaviors and mental health among Chinese college students. *Psychology Health & Medicine* **21**, 377-385, doi:10.1080/13548506.2015.1070955 (2016).

89 Yen, J. Y., Yen, C. F., Wu, H. Y., Huang, C. J. & Ko, C. H. Hostility in the real world and online: the effect of internet addiction, depression, and online activity. *Cyberpsychol Behav Soc Netw* **14**, 649-655, doi:10.1089/cyber.2010.0393 (2011).

90 Yu, X. Depressive symptoms and related factors analysis in english major college students. *Chinese Journal of Disease Control & Prevention* **15**, 145-148 (2011).

91 Yu, Y. *et al.* The role of family environment in depressive symptoms among university students: a large sample survey in China. *PLOS ONE* **10**, doi:10.1371/journal.pone.0143612 (2015).

92 Zeng, L. Study on depressed symptom and its influencing factors among sport professional college students. *Chinese Journal of School Doctor* **17**, 393-394 (2003).

93 Zeng, Q. *et al.* Analysis on influential factors and prevention way of depression among college students in a certain university. *Pratical Preventive Medicine* **13**, 1412-1413 (2006).

94 Zeng, Y., Wang, G., Xie, C., Hu, X. & Reinhardt, J. D. Prevalence and correlates of depression, anxiety and symptoms of stress in vocational college nursing students from Sichuan, China: a cross-sectional study. *Psychology Health & Medicine* **24**, 798-811, doi:10.1080/13548506.2019.1574358 (2019).

95 Zhai, D., Pan, X., Li, H., Mou, J. & Zhou, X. Survey on psychological and social factors of depression in medical college students. *Chinese Journal of Public Health* **21**, 12-13 (2005).

96 Zhang, F., Tang, Q., Deng, Y., Fan, H. & Wang, R. An analysis of mental health status of medical students and its related factors. *Chinese Journal of Clinical Psychology* **12**, 63-65 (2004).

97 Zhang, Q. Incidence rate of depression and related factors analysis in undergraduates of different crowd from normal university. *Chinese Journal of Clinical Rehabilitation* **9**, 92-93 (2005).

98 Zhang, Z., Wu, C., Liu, G., Fan, C. & Wang, H. Depression and the influencing factors of medical college students in guangzhou. *Chinese Journal of School Health* **27**, 51-52 (2006).

99 Zhang, W. *et al.* Exploratory study on relationship between life events and their influence on depressive status of undergraduates. *Chinese Journal of Behavioral Medicine and Brain Science* **24**, 374-377, doi:10.3760/cma.j.issn.1674-6554.2015.04.027 (2015).

100 Zhang, M. X., Mou, N. L., Tong, K. K. & Wu, A. M. S. Investigation of the effects of purpose in life, grit, gratitude, and school belonging on mental distress among Chinese emerging adults. *International Journal of Environmental Research and Public Health* **15**, doi:10.3390/ijerph15102147 (2018).
101 Zhang, G., Yang, X., Tu, X., Ding, N. & Lau, J. T. F. Prospective relationships between mobile phone dependence and mental health status among Chinese undergraduate students with college adjustment as a mediator. *Journal of Affective Disorders* **260**, 498-505, doi:10.1016/j.jad.2019.09.047 (2020).
102 Zhao, S. & Zhang, J. The association between depression, suicidal ideation and psychological strains in college students: a cross-national study. *Cult Med Psychiatry* **42**, 914-928, doi:10.1007/s11013-018-9591-x (2018).
103 Zheng, Y. & Zhou, Q. Study on mental health status of college student and its influential factors. *Modern Preventive Medicine* **35**, 4825-4827 (2008).
104 Zheng, S., Tong, Q. & Zheng, A. Investigation on depression and anxiety of college students and analysis of related factors. *Chongqing Medicine* **45**, 2835-2837, doi:10.3969/j.issn.1671-8348.2016.20.032 (2016).
105 Zhong, S., Tu, W., Shi, X. & Zhang, W. Study on related factors of depression in public security college students. *China Journal of Health Psychology* **19**, 1382-1384 (2011).
106 Zhou, R., Yang, C. & Pan, J. Investigation and analysis of depression and related factors among key medical college students in Guangzhou. *Journal of Neuroscience and Mental Health* **3**, 367-368 (2003).
107 Zhou, E. *et al.* Correlation between psychosomatic health and depressive symptoms among university students. *Journal of Medical Research* **38**, 50-54, doi:10.3969/j.issn.1673-548X.2009.10.018 (2009).
108 Zhou, H., Wan, D. & Liu, L. Mental health status and its correlation with life stress of postgraduates from agricultural universities in China. *Investigacion Clinica* **59**, 359-367 (2018).
109 Zhu, Z. *et al.* Frequency of breakfast consumption is inversely associated with the risk of depressive symptoms among Chinese university students: A cross-sectional study. *PLOS ONE* **14**, doi:10.1371/journal.pone.0222014 (2019).
110 Zong, J. G. *et al.* Coping flexibility in college students with depressive symptoms. *Health Qual Life Outcomes* **8**, 66, doi:10.1186/1477-7525-8-66 (2010).
111 Zou, F. *et al.* A study of psychological education on anxiety and depression of university students. *Chinese Journal of Clinical Psychology* **15**, 557-558 (2007).
112 Zou, P. *et al.* Associations between negative life events and anxiety, depressive, and stress symptoms: A cross-sectional study among Chinese male senior college students. *Psychiatry Research* **270**, 26-33, doi:10.1016/j.psychres.2018.09.019 (2018).
113 Zou, P. *et al.* Semen quality in Chinese college students: associations with depression and physical activity in a cross-sectional study. *Psychosomatic Medicine* **80**, 564-572, doi:10.1097/psy.0000000000000595 (2018).
